# Male-female communication enhances release of extracellular vesicles leading to high fertility in Drosophila

**DOI:** 10.1038/s42003-022-03770-6

**Published:** 2022-08-13

**Authors:** Javier Arturo Sanchez-Lopez, Shai Twena, Ido Apel, Shani Chen Kornhaeuser, Michael Chasnitsky, Andras G. Miklosi, Perla J. Vega-Dominguez, Alex Shephard, Amir Hefetz, Yael Heifetz

**Affiliations:** 1 Department of Entomology, The Hebrew University of Jerusalem, Rehovot, 76100 Israel; 2 Institute of Biochemistry, Food Science and Nutrition, The Hebrew University of Jerusalem, Rehovot, 76100 Israel; 3 ONI (Oxford Nanoimaging), Jordan Hill, Banbury Road, Oxford, OX2 8TA UK; 4 NanoView Biosciences, Malvern Hills Science Park, Geraldine Road, Malvern, WR14 3SZ UK; 5 DataGraph, Holon, 5880820 Israel

**Keywords:** Reproductive biology, Cell biology

## Abstract

The female reproductive tract (female-RT) must decipher the repertoire of molecular cues received from the male during copulation in order to activate and coordinate tract functionality necessary for high fertility. In Drosophila, this modulation is partially driven by spermathecal secretory cells (SSC). The SSC are a layer of cuboidal secretory glandular cells surrounding the spermatheca capsule where sperm is stored. It is unclear, however, how the SSC regulate the system’s activity. Here we show that mating activates the secretory machinery of the SSC. The SSC release a heterogeneous population of extracellular vesicles (EVs) which is involved in initiating and managing the increase in egg-laying, and possibly sperm storage. Moreover, sperm and male accessory gland proteins are essential for such mating-mediated SSC activity. Thus, mating regulates secretory/endocytic pathways required for trafficking of vesicles to SSC-female-RT target sites, which modulate and coordinate reproductive tract activity to achieve high fertility.

## Abstract (web summary)

Visualisation of spermathecal secretory cell (SSC) secretion in Drosophila reveals that the SSC produces extracellular vesicles to modulate and coordinate reproductive tract activity.

## Introduction

Interactions between male and female, from pre-mating through post-mating, shift female physiology to accommodate high fertility. In many species, including insects, male-female communication involves a complex combination of cues at multiple modalities that affect female physiology by manipulating female control of reproduction. Among these cues are signals transferred with sperm to the female in the seminal fluid, during copulation^1–3^. In response to these cues, and together with the paternal contributions, the female reproductive tract (female-RT) coordinates the formation of an environment promoting gamete production, transport, maintenance and union, leading to healthy offspring^4–9^. The manner in which different combinations of input modalities and output networks lead to high fertility remains largely unresolved.

**Fig. 1:**
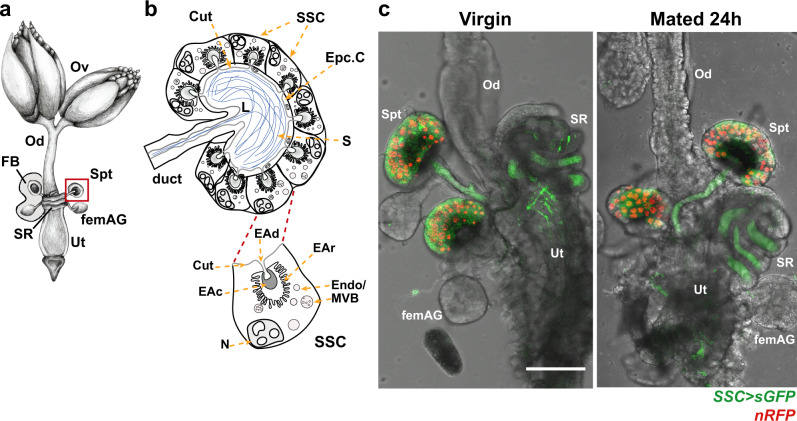
Secretions produced by the spermathecal-secretory cells (SSC) reach the seminal receptacle and uterus. **a** Schematic of the female reproductive tract (female-RT; adapted from Avila, F.W. et al.^6^). The female-RT is composed of ovaries (Ov), oviduct (Od), seminal receptacle (SR) and a pair of spermathecae (Spt), where sperm is stored, a pair of female accessory glands (femAG), and a uterus (Ut). **b** Schematic of spermatheca (red square in **a**) displaying the spermatheca secretory cells (SSC) radially organized around a lumen (L) where sperm (S) is stored. The lumen is lined by dark chitin cuticle (Cut) and epicuticular cells (Epc.C). Each SSC displays a nucleus (N), endosomes (Endo), multivesicular bodies (MVB) and an end apparatus (EA), a specialized structure formed by a reservoir of convoluted membrane (EAr), a cuticular porous cavity (EAc) and a duct (EAd) through which secretions are secreted to the lumen. **c** Representative confocal maximum projection images of the female -RT that specifically expressed the secreted form of GFP (Send1 > sGFP, here after SSC > sGFP) in the SSC. The nuclei of the SSC are observed as nRFP (scale bar = 100 µm).

In *Drosophila melanogaster*, formation of the female-RT environment is partially driven by spermathecal secretory cells (SSC), a layer of cuboidal secretory glandular cells that surrounds the spermatheca capsule where sperm is stored, and by the female accessory glands (femAG)^6,10,11^ (Fig. 1a, b). Simultaneous disruption of spermatheca and femAG development results in female sterility^12^. In addition, sperm storage in the spermatheca requires the expression of *Hormone receptor-like in 39* (*Hr39*, a nuclear hormone receptor and a master regulator of gland development) and canonical secretions from both glands. The transit of eggs through the female-RT requires *Hr39* expression and non-canonical secretions^13^. Furthermore, ablation of the SSC after female eclosion showed that SSC function is required for sperm to reach the spermatheca and retain motility while in storage, and for proper egg-laying^14^. It is unclear how the SSC regulate these activities, but their effect on the female reproductive system is pronounced, as any modification in their function leads to a reduction in fertility. One possible mechanism by which the SSC regulate processes in other organs is via secretion of soluble factors and/or extracellular vesicles (EVs) into the reproductive tract environment^4^. EVs, a heterogeneous family of cell-derived vesicles, are released by most cell types and can elicit a phenotypic response in the secreting cell itself, or in neighboring and/or distant target cells. EVs can emerge from the plasma membrane (microvesicles) or be derived from endosomal compartments and formed within multivesicular bodies (MVBs) (exosomes). Given that EVs shuttle lipids, proteins, RNA, DNA, metabolites and other components, they can change the fate of recipient cells^15^. In Drosophila, the male accessorygland secretory cells produce and secrete EVs which are transferred to the female during mating and inhibit her re-mating behavior^16^. Whether EVs are used by the SSC to modulate and coordinate female-RT activity to achieve high fertility, and what roles the male plays in such SSC-female-RT communication, is unknown.

Here we use Drosophila female-RT as a model to study how cues exchanged between males and females translate to high fertility. Specifically, we focused on SSC-female-RT intercellular communication and set out to clarify whether these interactions are modulated by EVs and mediated by male components. We utilized Drosophila genetic tools to visualize, characterize and reveal possible mechanisms by which the SSC regulate processes in the female-RT. We show that SSC-derived secretions target various regions of the female-RT, and we provide the first evidence that SSC use EVs to modulate and coordinate reproductive tract activity. Our data furthermore show that SSC secrete a heterogeneous EV population enriched with exosomes, and that the secretory/endocytic activity of the SSC is mating-responsive. Our results highlight the involvement of SSC-derived EVs in regulating egg-laying, and we discuss their potential role in the egg-laying process. The involvement of EVs in SSC-female-RT intercellular communication adds an entirely new layer of regulation towards the achievement of functional female-RT and high fertility, about which little is known in Drosophila and other organisms.

## Results and discussion

### Cues delivered to the female-RT post-mating modify the secretory activity of the SSC

The SSC have a characteristic secretory morphology with an end apparatus reservoir (EAr) that collects and dispatches secretion into the spermatheca lumen^17–19^ (Fig. 1a, b; supplementary Fig. 3a). Very little is known about the composition and functions of spermathecal secretions. To investigate whether the SSC secretion reaches other regions of the female-RT, we first expressed a secretory form of GFP (sGFP)^20^ specifically in the SSC using Send1-GAL4^14^, and visualized the route of SSC-derived secretions in the female-RT. sGFP was detected inside the SSC, spermatheca lumen and ducts, the seminal receptacle and the uterus of both virgin and mated females (Fig. 1c), indicating that SSC-derived secretions target various regions of the female-RT. GO term enrichment analysis of genes expressed in the spermatheca^21–25^ revealed that genes associated with “endosome transport via multivesicular body” and “exocytosis” are significantly enriched in the spermatheca as compared with the whole body (*p* = 0.0002, *p* = 0.00008 respectively; Supplementary Fig. 1). Interestingly, mating increases the enrichment of genes associated with “exocytosis” at 3 h post-mating, and of genes associated with “vesicle-mediated transport to the plasma membrane” and “endocytosis” at 3 and 6 h post-mating, in contrast with virgin females; this suggests that mating regulates secretory pathways and activity of the SSC (Supplementary Fig. 1). Some members of the Ras-related in brain (Rab) family of GTPases, which are regulators of core secretory and endocytic machinery, were highly enriched in the spermatheca relative to their expression in the whole body (e.g. *Rab35, Rab5, Rab7*). Furthermore, the expression level of some of these Rabs (e.g. *Rab7, Rab11*) is significantly changed post-mating (*p* < 0.05; Supplementary Fig. 2a).

**Fig. 2:**
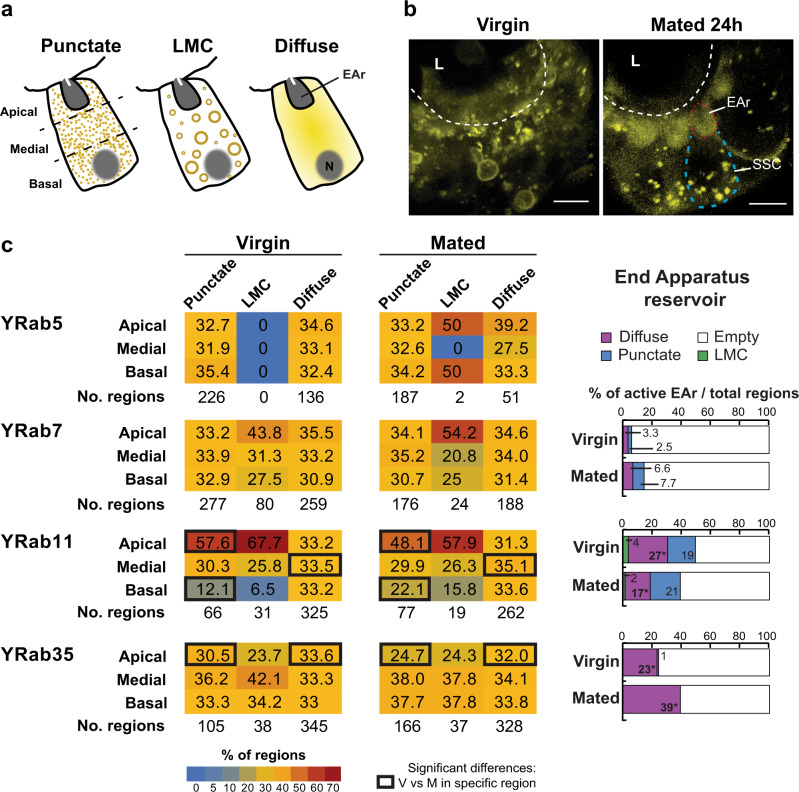
The subcellular localization of Rab GTPases in the SSC is regulated by mating. Flies that express endogenous YFP-tagged Rabs (*YRab*; *YRab5*, *YRab7*, *YRab11*, and *YRab35*) were used to visualize expression and determine subcellular localization of Rabs in the SSC. **a** Schematic depicting the type of localization patterns of YRabs in the SSC based on Dunst et al.^26^ (punctate, large membranal compartment LMC or diffuse) within the apical, medial, basal subcellular regions of the SSC or in the End Apparatus reservoir (EAr); N = nuclei. **b** Representative images that show YRab35 in unmated and mated SSC at 24 h post-mating. Red and blue broken lines depict the EAr at the apical region of the SSC, respectively; L- lumen; scale bar = 5 µm. **c** Heatmap profiles showing the distribution of different YRabs in SSC of virgin and mated females at 24 h post-mating. The heatmap color code represents the percentage of YRabs expressed in each subcellular region (apical, medial, basal), for each of the three pools, in every radial sections of virgin and mated SSC (the number of regions in which a specific YRabs was expressed is shown at the bottom of each YRab heatmap). The bars at the right side represent the percentage of active EAr (i.e. EAr that contain YRab) in SSC of virgin or mated females out of the total sections observed (note, YRab5 was never observed in the EAr). Significant differences between virgin and mated (*n* = 15–30 spermathecae from 10–20 flies) were estimated by the Incident Risk Ratio (IRR), indicated by black squares that surround cells in the heatmap and * inside the bar graphs (*p* < 0.05). See also Supplementary Fig. 2.

Given the primary role of Rab GTPases as regulators of membrane trafficking, we next used YRab fly lines^26^ to visualize the expression and subcellular localization of Rab5, Rab7, Rab11 and Rab35 in SSC of virgin and mated females. We divided the spermatheca into 8 radial sections (Supplementary Fig. 2b) and followed the terminology of Dunst et al. to describe the appearance and analyze the intracellular distribution of the different YRab proteins in the SSC^26^. YRabs expressed in the SSC are associated either with large membranal compartments (LMC), small intracellular membranal punctate (punctate), or with non-membrane-associated pools or small vesicles below the resolution of the light microscope (diffuse) (Fig. 2a, b). Since each YRab can exist in different pools within the cell (LMC, punctate, diffuse), we examined whether or not the YRabs were expressed in each subcellular region (apical, medial, basal), for each of the pools, in every radial section of virgin and mated SSC (Fig. 2a, c). In addition, we determined whether each of the YRabs is enriched in a specific subcellular region, for each of the pools, in every radial section of virgin and mated SSC (Supplementary Fig. 2c). Although the end apparatus reservoirs are found within the apical subcellular region, the reservoirs were given special attention as they are independent membranous structures that might inform which of the YRabs is necessary for release of secretions to the spermatheca lumen in virgin and mated states. We found that mating alters the enrichment of YRabs and changes the distribution of some YRabs toward the apical and/or basal subcellular regions of the SSC (Fig. 2c and Supplementary Fig. 2c). In virgin females, YRab5 and YRab7 are distributed throughout the cytosol of the SSC. Mating has no significant effect on YRab7 and YRab5 distribution in the SSC (Fig. 2c). In contrast, YRab11 in punctate pools is polarized apically in virgin SSC. At 24 h post-mating, YRab11 is polarized apically, but the apical punctate pool is significantly decreased; there are thus fewer regions where YRab11 can be detected at the apical subcellular region (YRab11 was found in 57.6% of the SSC regions in virgin females vs. 48.1% of the regions in mated females; *p* < 0.05). Additionally, mating significantly increased YRab11 punctate and diffuse pools at the basal and medial subcellular regions, respectively (12.1 and 33.5% of the SSC regions vs. 22.1 and 35.1% of the regions post-mating; *p* < 0.05; Fig. 2c). Conversely, punctate and diffuse pools of YRab35 were distributed throughout the cytosol of the virgin SSC, while these pools are significantly decreased post-mating at the apical subcellular regions (punctate and diffuse pools: 30.5 and 33.6% of the regions in virgin vs. 24.7 and 32% of the regions in mated; respectively, *p* < 0.05; Fig. 2c). Interestingly, the percentage of SSC with YRab35-associated diffuse pools detected in the end apparatus reservoirs is significantly increased post-mating, while YRab11-associated diffuse pools are decreased post-mating (23 vs. 39% SSC; 27 vs. 17% SSC; respectively, *p* < 0.05; Fig. 2c). These results indicate that in SSC of virgin and mated females, YRab11 punctate pools are apically polarized, while the end apparatus reservoirs contain more YRab35-associated diffuse pools, post-mating.

Finally, the overall abundances of YRab5 and YRab7 associated with punctate pools, and YRab5, YRab7, YRab11 and YRab35 associated with diffuse pools, were significantly different post-mating (*p* < 0.05; Supplementary Fig. 2c). Furthermore, the number of regions in which YRab5 and YRab35 associated with punctate pools are highly abundant, significantly increased post-mating in the medial subcellular region (4 vs. 26% of the SSC, and 5 vs. 17% of the SSC, respectively; *p* < 0.05; Supplementary Fig. 2c). In contrast, the number of regions in which YRab5 associated with diffuse pools is highly abundant, significantly decreased post-mating throughout the SSC (*p* < 0.05; Supplementary Fig. 2c).

Rab11 and Rab35, which act in the endosome recycling pathway^27,28^, also regulate exosome secretion in flies^29,30^ and other organisms^28^. Rab35 has been found to mediate endocytosis recycling after cargo internalization^31,32^. Taken together, these findings suggest that mating changes the SSC secretory/endocytic activity at the apical subcellular region (towards or away from the spermatheca lumen). Changes in the distribution of Rabs localized in the apical or basal region were shown to be characteristic of the transition from early egg chamber follicle cells to later egg chamber follicle cells^26^. Different distributions of Rabs in the SSC could plausibly reflect a transition from the terminal differentiation and final development of the female-RT that characterize the first 24 h post-mating, to a functional female-RT^33^ which requires, for example, components secreted from mature SSC to maintain sperm storage and support a high rate of egg-laying.

### Mating enhances the secretory/endocytic activity of the SSC

**Fig. 3:**
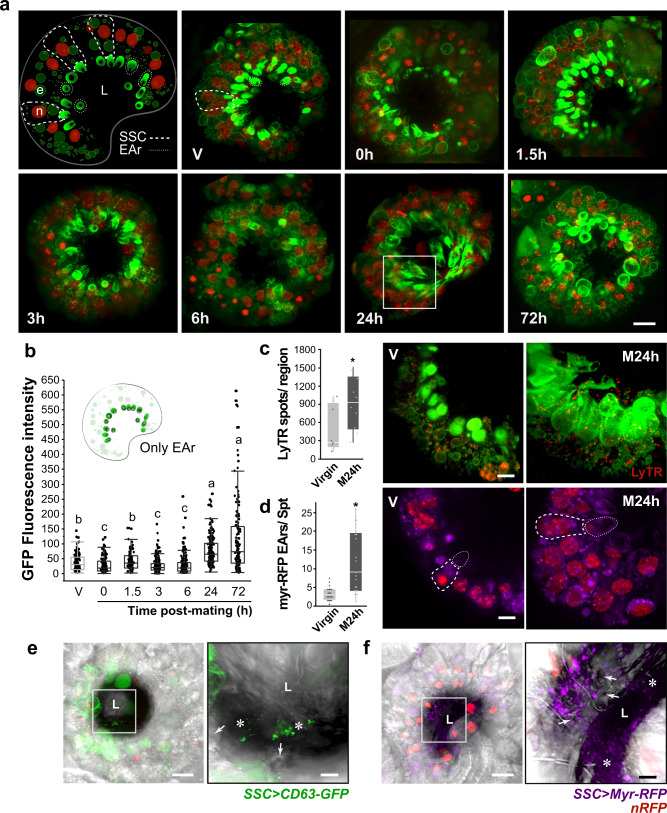
Mating modulates the secretory activity of the SSC. **a** Representative confocal maximum projection images of CD63-GFP expressed in the SSC of virgin and mated females. Top left: schematic that represents one spermatheca slice that shows the structural composition of the spermatheca: SSC (dotted outline), CD63-GFP (green) which is localized to cellular compartments such as endosomes (e) and the end apparatus reservoir (EAr); spermathecae lumen (L); nuclei (n, nRFP); (see also Fig. 1 and Supplementary Fig. 3 for the morphology of virgin spermatheca, Supplementary movie 1  time lapse of virgin and mated SSC at 72 h post-mating, and Supplementary movie 2 that highlights an endosome containing CD63-GFP-positive puncta, described before as active intraluminal vesicles^16^). **b** CD63-GFP fluorescence intensity level of individual EAr (see also Supplementary Fig. 3; 10 EAr per spermatheca of *n* = 15–25 spermathecae from 8–15 flies); Letters denote significant differences (one-way ANOVA, with multiple comparison post-hoc test, *P* < 0.05). **c** Box plot of the number of LysoTracker deep red (LyTR) puncta found in virgin and mated SSC at 24 h post-mating; also shown, localization of CD63-GFP and LyTR in representative regions of the spermatheca as exemplified in the insert of panel 1a at 24 h post-mating. **d** Number of myristoylated (myr)-RFP positive EArs of virgin and mated females at 24 h post-mating, and representative spermathecae that express myr-RFP (purple). Broken lines denote SSC and EAr; nuclei nRFP. Box plots represent the distribution of 15–20 spermathecae per condition from 8-12 flies. *p < 0.0001, one-way ANOVA. Overlay of **e** CD63-GFP or **f** myr-RFP fluorescence and bright-field micrographs; insets denote the magnified regions that follow (See also Supplementary movies 3 and 4). Stars = presence of CD63-GFP or myr-RFP-positive secretions. Arrows = pores in the cuticle. Scale bars of **a**, **e** and **f** = 20 µm, **c** and **d** = 10 µm, insets in **e** and **f** = 5 µm. The box plots represent maximum, median and minimum values with outliers.

Our initial analyses indicated that SSC-derived secretions target various regions of the reproductive tract, and that the SSC are enriched in secretory pathways involved in EV secretion. Therefore, we next examined whether the SSC produce and secrete EVs. To visualize EVs, we specifically expressed in the SSC GFP-tagged CD63, a mammalian tetraspanin, a superfamily of proteins that organize membrane microdomains and are highly enriched in EVs^16,34^. In SSC of virgin females, CD63-GFP was localized in endosomes and accumulated in the end apparatus reservoir (see Fig. 1b; Supplementary Fig. 3a; Fig. 3a). CD63-GFP fluorescence levels decreased significantly in the cytoplasmic region and end apparatus reservoir immediately after mating (0 h, *p* < 0.05; Supplementary Fig. 3b; Fig. 3b, respectively). Over the following 72 h, we observed oscillating levels of CD63-GFP in the end apparatus reservoir. CD63-GFP fluorescence level is high by 1.5 h, then low at 3–6 h, only to increase at 24 h and peak at 72 h post-mating (Fig. 3a, b; Supplementary Movie 1). A similar oscillation was detected in the size of the end-apparatus reservoir (Supplementary Fig. 3d, e), indicating that mating mediates dynamic changes in the amount of secretion in the end apparatus reservoir. CD63-GFP-positive vesicles were found in the spermatheca lumen at 24 h post-mating (Fig. 3e, Supplementary Movie 2). Mating also increased the number and volume of acidic compartments found in the SSC at 24 h post-mating, shown as intracellular compartments stained with LysoTracker Red^35^, (Fig. 3c). Increased intravesicular pH may reflect elevated endocytic activity, for processing of mating-derived components, for example. The mating-induced enlargement of the LysoTracker-positive spots in the SSC plausibly suggests an increase of late endosomes/MVB^34^, a process that requires acidification^35^. To verify the mating effect detected upon expressing CD63-GFP in the SSC, we expressed in the SSC a myristoylated RFP (myr-RFP) co-translational modification that targets proteins to membranes^36^. The myristoylation consensus sequence fused to RFP labeled intracellular membranes in the SSC and as a consequence, EVs derived from these labeled membranes are tagged by myr-RFP^36,37^. In SSC of virgin females, myr-RFP localized only slightly to the end apparatus reservoir and mating significantly increased the number of end apparatus reservoirs with myr-RFP (*p* < 0.0001; Fig. 3d). myr-RFP-positive vesicles were also detected in the spermatheca lumen, indicating that they are secreted from the end apparatus reservoirs (Fig. 3d, f and Supplementary Movie S3). Corrigan et al.^16^ showed that sperm transferred to the female during copulation carry CD63-GFP-derived exosomes secreted from the secondary cells of the male-accessory gland. They found that sperm and exosomes accumulate in the anterior uterus at 20 min after the onset of mating^16^. Although Corrigan et al. did not detect CD63-GFP signals in the sperm storage organs, it is possible that sperm that enters the spermatheca lumen, has male exosomes docked onto its membrane, in addition to the other mating-derived signals received by the SSC. We propose that the SSC can actively take up and process these exosomes, non-exosomal seminal components and other signals required for sustaining the post-mating effect.

**Fig. 4:**
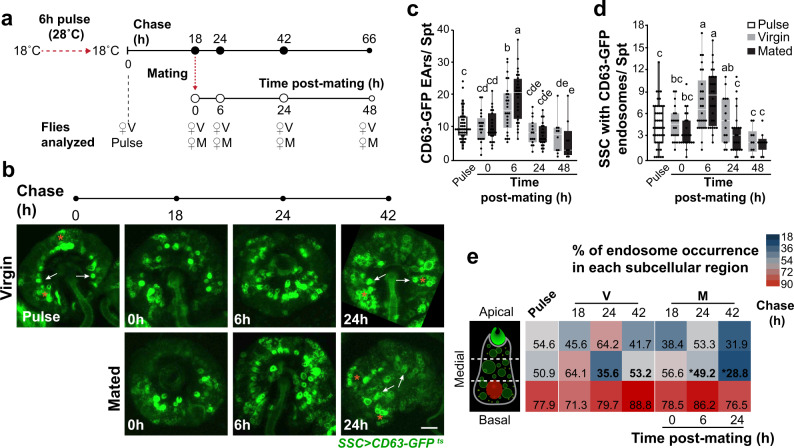
Mating changes the CD63-GFP trafficking dynamics in the SSC. **a** Schematic that describes the pulse-chase experimental design: pulse duration, CD63-GFP chase time (full circles), time of sampling virgin (V) and mated (M) females (empty circles). **b** Representative confocal maximum projection images of virgin and mated spermatheca expressing CD63-GFP; arrows and stars denote SSC with CD63-GFP-positive EAr and endosomes, respectively. Scale bar = 20 µm. **c** Average number of CD63-GFP-positive EAr in the spermatheca at different times post pulse and mating. **d** Average number of SSC with CD63-GFP-positive endosomes in the spermatheca at different times post pulse and mating (see also Supplementary Fig. 4a, c). Bars represent the mean ± SE, *n* = 10–28 spermathecae from 7–17 flies per condition; letters denote significant differences analyzed by two-way ANOVA and Tukey HSD multiple comparison post-hoc test *p* < 0.05. **e** Heatmap depicting the percentage of endosomes detected in different subcellular regions (apical, medial or basal) within the SSC of virgin or mated females at different time post-mating (Supplementary Fig. 4b). Stars and bold numbers in the heatmap highlight significant difference (one-way ANOVA, *p* < 0.05).

We next used a pulse–chase approach to assess whether mating affects trafficking and degradation/release of CD63-GFP through the secretory and endosomal systems. A short pulse of CD63-GFP expression in the SSC (6 h, at 28 °C) was followed by a chase period of 66 h (18 °C) during which a subgroup of females was also mated at 18 h post-chase (Fig. 4a). At the end of the pulse/start of chase we detected CD63-GFP-positive end apparatus reservoirs and endosomes in several SSC (Fig. 4b–d), which were possibly active at that time (Fig. 4c). Mating significantly increased the number of CD63-GFP-positive end apparatus reservoirs (6 h post-mating, *p* < 0.05; Fig. 4c) and end apparatus area (6–24 h post-mating; *p* < 0.05; Supplementary Fig. 4c). The number of SSC-containing CD63-GFP endosomes significantly decreased at 24 h post-mating, indicating a mating-induced release / usage of CD63-GFP (*p* < 0.05, Fig. 4d; Supplementary Fig. 4a). Notably, mating also changed the spatial distribution of endosomes in the SSC; the percentage of endosomes at the medial subcellular region significantly increased at 6 h and decreased at 24 h post-mating in comparison with virgin females (*p* < 0.05; Fig. 4e). Furthermore, analysis of endosome distribution patterns along the SSC revealed that mating significantly increased the percentage of SSC in which the CD63-GFP-marked endosomes were widely distributed at 6 h and reduced the percentage of SSC with CD63-GFP-marked endosomes at the apical subcellular region at 24 h post-mating (*p* < 0.05; Supplementary Fig. 4b). The pulse-chase results indicate that SSC synthesize CD63-GFP, which traffics on endosomes, plausibly to other vesicular compartments and the end apparatus reservoir. Mating enhanced the rate of CD63-GFP trafficking and changed the spatial distribution of endosomes in the SSC. The significant reduction in the number of GFP-positive SSC at 24 h post-mating, and the increase in size of the end apparatus reservoir with time after mating may indicate trafficking toward the apical region for release or storage in the end apparatus reservoir. Based on these results, we hypothesize that mating modulates SSC endosomal trafficking and supplies exogenous material which may modulate processes within the SSC that are necessary for high fertility.

### SSC-derived EVs are a heterogeneous population enriched with exosomes

**Fig. 5:**
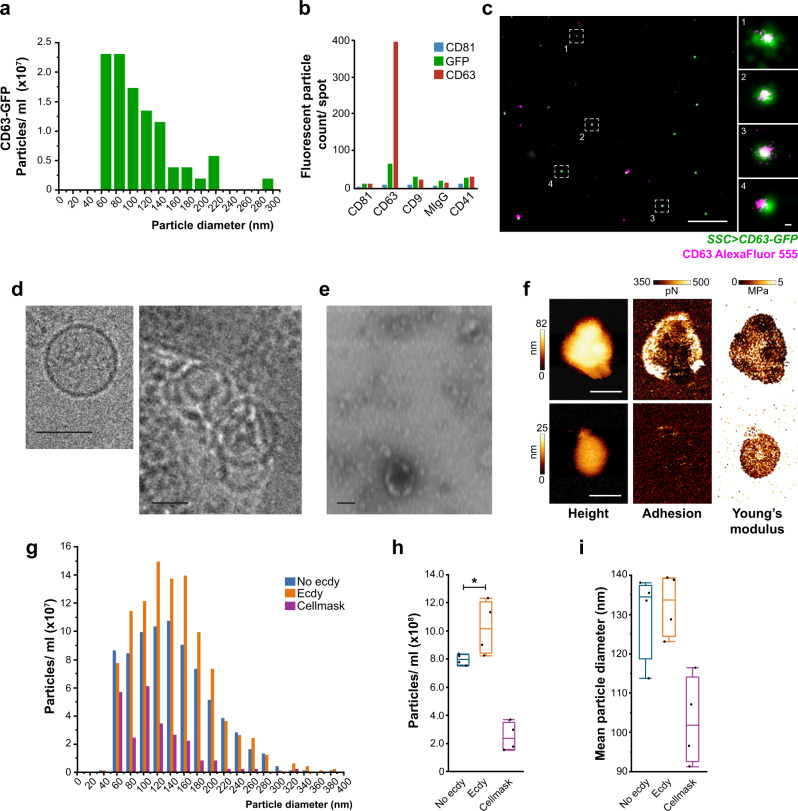
The spermatheca releases EVs with specific characteristics. **a**–**e** The spermathecae (SSC > CD63-GFP) were cultured ex vivo to characterize the particles that were released to the spent media: **a** size distribution of CD63-GFP-positive particles (*n* = 25 spermathecae from 15 flies) using single-particle tracking of the Nanoimager. **b** Characterization of CD63-GFP particles by ExoView (100 spermathecae from 50-60 flies) using anti-GFP or anti-CD63. **c** Representative dSTORM image of EVs found in the spent media of SSC expressing CD63-GFP (*n* = 25 spermathecae from 15 flies), stained with AlexaFluor555 conjugated anti-CD63 primary antibody. The image shows CD63-GFP-positive EVs and single CD63 AlexaFluor555 molecules the EV’s surface. Scale bars = 1 µm and insets = 20 nm. **d**–**e** The morphology of EVs in the spent media was observed by **e** negative staining in STEM; scale bar = 200 nm; and by **d** Cryo-TEM; Scale bar = 100 nm (see also Supplementary Fig. 5a-c). **f**–**i** The spent media of ex vivo cultured spermathecae were analyzed for the presence of EVs: **f** Representative AFM scans of SSC-EVs isolated by acoustic sorting (*n* = 100 spermathecae from 70 flies), showing from left to right: height (nm), adhesion (pN) and Young’s modulus images (MPa). Scale bars = 100 nm (see Supplementary Fig. 5d–g). **g**–**i** Single-particle tracking in the nanoimager. **g** CellMask^TM^-positive particle size distribution and concentration profiles of EVs in the spent media of spermathecae incubated in media alone (No ecdy) or with ecdysone (Ecdy) (see also Supplementary Movie 5 for a time lapse of spermathecae end apparatus and endosomal activity post-ecdysone stimulation and methods, ex vivo *spermatheca culture* section); media with only CellMask^TM^ stain served as a control for the formation of dye aggregates. **h** Particle concentration and **i** mean particle diameter (nm) from **g**; Box plots are the measurements of particles from four frames of spent media from 25 spermathecae; boxes represent maximum, median and minimum values with outliers; one-way ANOVA, with nonparametric Wilcoxon multiple comparison post-hoc test; *p < 0.0001.

To confirm that the SSC secrete EVs, we next developed an ex vivo system that enabled us to culture the whole spermatheca and collect spent media for analysis. Using the nanoparticle tracking mode of the ONI Nanoimager, we found that SSC secrete EVs to the media (Fig. 5a, c). The size of SSC-derived CD63-GFP EVs released by the SSC was heterogeneous, ranging between 60–220 nm; their average diameter was 104 nm (Fig. 5a). Release of SSC-derived CD63-GFP EVs to the media was also confirmed with ExoView™ using antibodies to probe CD63 and GFP on single EVs and confocal microscopy (Fig. 5b; Supplementary Fig. 5a). Furthermore, dSTORM (ONI) imaging enabled remarkably precise visualization of CD63-GFP colocalization, and of spatial organization of single CD63 molecules on SSC-derived EV surfaces (Fig. 5c). To further verify that the SSC-derived CD63-GFP EVs released from the spermatheca and detected in the spent media are indeed EVs, we performed negative staining (visualized by scanning transmission electron microscope, STEM) and Cryogenic Electron Microscopy (Cryo-EM) (Fig. 5d, e; Supplementary Fig. 5b, c). Visualization by STEM revealed that the particle-like vesicles exhibit various sizes of typical lipid bilayer spherical cup-shaped morphology (Fig. 5e, Supplementary Fig. 5b). The Cryo-EM added another layer of information on the vesicles regarding their structure, membrane, and lumen. The visualized vesicles showed the clear presence of a lipid bilayer and appeared to be filled with electron-dense material which gave their lumen a darker texture. In addition, most of the vesicles were intact and had a round shape, and some were non-spherical and elongated in shape (Fig. 5d; Supplementary Fig. 5c). Most notably, a similar pattern of SSC-derived EVs released to the media analyzed by CellMask™ (i.e. CellMask-positive EVs) was more heterogeneous, ranging between 60–280 nm (Fig. 5g); their average diameter was 135 nm (Fig. 5i). Interestingly, the nanomechanical properties of individual SSC-derived EVs, analyzed by Atomic Force Microscopy (Supplementary Fig. 5d–g; Fig. 5f), showed that the vesicles’ centers are softer than the peripheral areas, which are more adhesive (Fig. 5f). The small EVs (45–70 nm) exhibited higher stiffness (300 ~ 600 kPa) and adhesion (400 ~ 550 pN) than the larger EVs (Supplementary Fig. 5f, g); this was also the case for human cancer cell small EVs and exomeres, which displayed greater stiffness (145 ~ 816 MPa) than larger cancer-derived EVs^38^. Anselmo et al. used hydrogel nanoparticles with a wide range of elasticity (0.255–3000 kPa) to identify the effect of elasticity on endocytosis, phagocytosis, targeting, and circulation of particles in the blood. They showed that soft nanoparticles are able to persist in higher numbers and for a shorter time in the circulation compared to stiffer nanoparticles, and that stiffer nanoparticles were more bound/internalized than the softer nanoparticles. They also found differences in targeting: soft nanoparticles target the spleen and lungs more than their stiffer counterparts^39^. The above findings collectively define for the first time, in Drosophila, the unique signature properties borne by spermatheca EVs. The population is composed primarily of stiffer particles in the size-range of exosomes (60–100 nm) and also contains particles in the size-range of microvesicles (120–220 nm). Additionally, these findings led us to hypothesize that spermatheca-EVs may last for a short time in the female-RT environment and are highly internalized by their targets. Interestingly, human vesicles are much stiffer than those of Drosophila; whether this difference highlights variation in the EVs of different organisms, indicates that softer EVs can circulate more easily through the smaller Drosophila body, or is connected to other characteristics of uptake, requires further investigation.

### SSC-derived EVs are involved in modulating fecundity and egg-laying rate

**Fig. 6:**
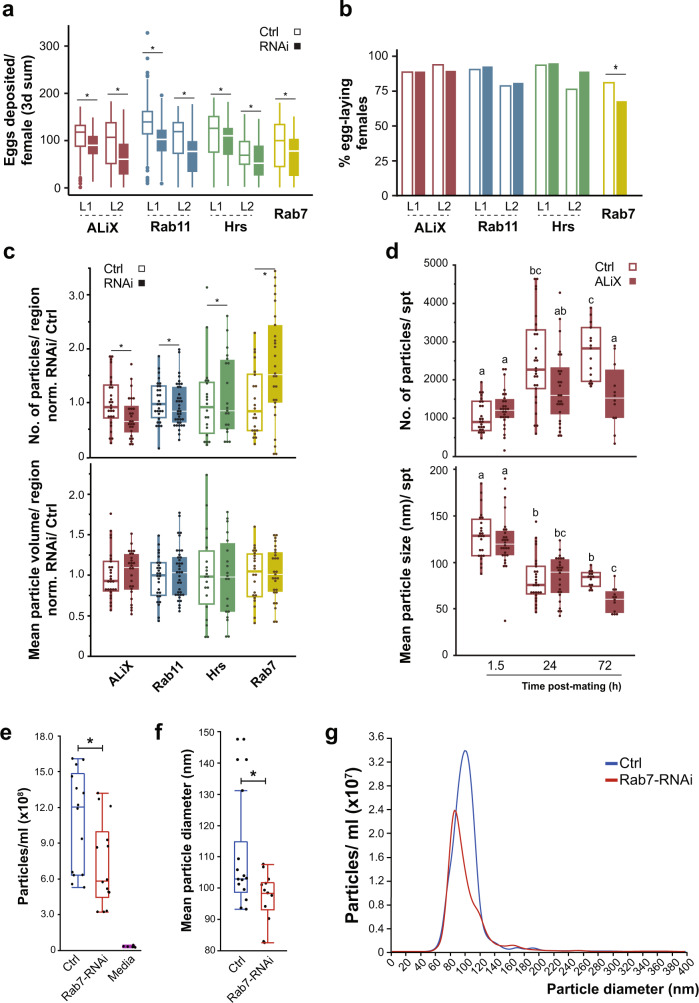
Interfering with *ALiX*, *Rab11*, *Hrs* and *Rab7* in the SSC impairs fecundity, affects SSC-CD63-GFP trafficking. **a**, **b** *ALiX*, *Rab11*, *Hrs* and *Rab7* were silenced specifically in the SSC (Control females—no RNAi, open box; RNAi expressing females, full box), and we evaluated different parameters associated with fly fecundity: **a** number of eggs deposited per female (only egg-laying females) in 3 days (box plots) and **b** the percentage of females that deposited eggs. Boxes represent maximum, median and minimum values with outliers and bars represent the percentage of egg-laying females (50–150 females per condition of four biological replicates). L1 and L2 represents the two lines examined. Egg count distribution was analyzed using a zero-inflated Poisson and logistic regression models. Egg deposition rate of the egg-laying females was analyzed by one-way ANOVA *p < 0.05; **p < 0.01; ***p < 0.001 (see Supplementary Fig. 6 for the extended evaluation of egg-laying). **c**, **d** RNAi for *ALiX*, *Rab11*, *Hrs* and *Rab7* was co-expressed with CD63-GFP in the SSC. We evaluated in each region **c** the intracellular CD63-GFP pool as the number of CD63-GFP particles (top) and their mean size (bottom) of RNAi normalized to their respective controls at 24 h post-mating. Boxes represent maximum, median and minimum values with outliers of 20–37 females per condition (see Supplementary Fig. 7 for representative images of SSC co-expressing CD63-GFP with different RNAi lines). Differences between RNAi and control were analyzed by GLM with Poisson distribution followed by a post-hoc pairwise comparisons with Bonferroni correction (*p < 0.05). **d** The total number of CD63-GFP particles per region (top) and their mean size (bottom) in control and silenced (empty and full boxes respectively) *ALiX*-RNAi SSC at 1.5, 24 and 72 h post-mating. Boxes represent maximum, median and minimum values with outliers of 25–37 spermathecae per condition (From 11–19 flies); letters denote significant differences (two-way ANOVA and Tukey HSD multiple comparison post-hoc test, *p* < 0.05). **e**–**g** The spermathecae of control (blue) and *Rab7-*RNAi flies (red) were cultured ex vivo. The spent media was analyzed by NTA to determine **e** particle concentration, **f** mean particle diameter (nm) and, **g** size/concentration profile of particles released by control or *Rab7-*RNAi spermathecae. Box plots show data of spent media from 150 spermathecae per condition of three biological replicates (100 flies per replicate); boxes represent maximum, median and minimum values with outliers. The total number and mean size of particles released by either Control or Rab7-RNAi were compared by one-way ANOVA; *p < 0.0001.

To determine whether SSC-derived EVs are involved in modulating female fertility, we silenced the expression of *ALiX*, *Hrs, Rab11* and *Rab7*, in the SSC^16,40,41^. Hrs (Hepatocyte growth factor regulated tyrosine kinase substrate) is a component of the ESCRT-0 complex required for the sorting of ubiquitinated proteins into MVBs^40,42,43^. As part of the endosomal maturation process, Hrs has been shown to have a role in ESCRT-dependent exosome biogenesis and secretion in mammals and flies^16,34,40,42–44^. ALiX (ALG-2–interacting protein X) is a multifunctional adapter protein involved in cell adhesion, membrane protein sorting and recycling, and apoptosis^45^. ALiX interacts with several ESCRT complex proteins associated with membranes, where it facilitates receptor recycling, and is associated with endosome protein sorting^45,46^. ALiX is required in exosome biogenesis in mammalian cells and flies, where it induces invagination of the endosomal membrane for the formation of ILVs^16,46,47^. Rab11 is localized in post-Golgi vesicles that facilitate sorting of membranal and secretory proteins in endosomes tethered to the plasma membrane^48^, and as part of vesicle recycling, Rab11 has been implicated in exosome secretion^27,29,30^. Rab7 is involved in the progression of early to late endosomal trafficking^49^ and the delivery of cargo from MVB to the lysosome^50^. It is also involved in secretion by Drosophila male-accessory gland secretory cells^16^ and regulates exosome secretion in MCF7 cells^47^. Silencing each gene significantly decreased the number of eggs deposited during the first 3 days post-mating (*p* < 0.05; Fig. 6a) but had no effect on hatchability (i.e. ratio of adults/eggs; see supplementary data 2). Each of the genes had a unique effect on egg-laying (Supplementary Fig. 6). While *ALliX*- and *Hrs*-RNAi females lay fewer eggs than control females 48–72 h post-mating (*p* < 0.001; *p* < 0.01, respectively), *Rab11*-RNAi and *Rab7*-RNAi females also exhibit reduced egg-laying at 8 days post-mating (*p* < 0.001). Moreover, *ALiX*- and *Rab11*-RNAi females showed a significant delay in initiating egg-laying (*p* < 0.0077; *p* < 0.0002, respectively), and the number of ovipositional females was significantly lower than in controls at 6 h and 8 days post-mating (*p* < 0.001; *p* < 0.05, respectively) (Supplementary Fig. 6). Thus, proper function of *ALiX*, *Hrs*, *Rab11* and *Rab7* in the SSC is needed to maintain the short- and long-term egg-laying rate. These results are consistent with the findings of Schnakenberg et al., who showed that SSC ablation reduces egg-laying and egg-laying rate, with no effect on fertility^14^, as well as the findings of Sun and Spradling, who demonstrated that SSC secretions are required for proper egg-transit through the female-RT^13^.

**Fig. 7:**
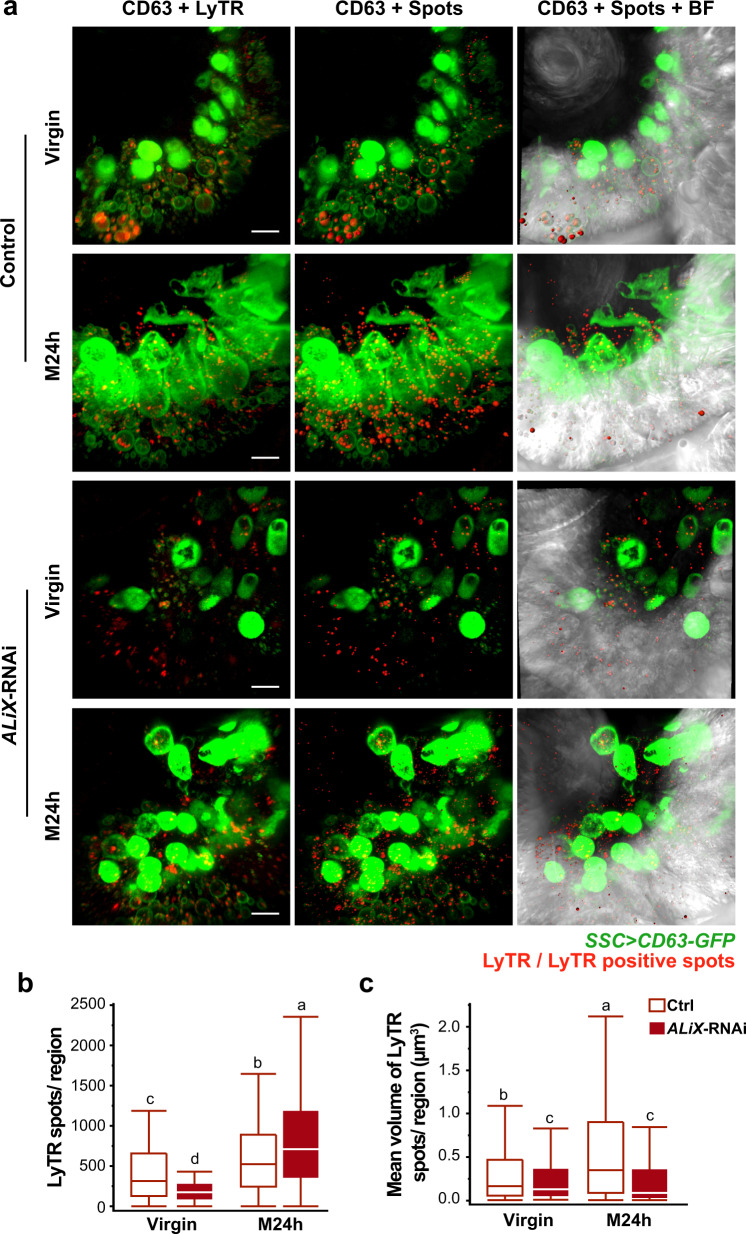
Silencing *ALiX* in the SSC changes the pattern, number and size of acidic compartments. **a** Representative sections of the spermatheca (the type of sampled region is shown in the inset of Fig. 3a) of control (no RNAi) or *ALiX*-RNAi virgin and mated at 24 h post-mating (M24 h) females. The micrographs depict SSC expressing CD63-GFP stained with LysoTracker Deep Red (LyTR, left), SSC with LyTR spots rendered as plastic surfaces (middle) and rendered LyTR spots overlaid on the bright field (BF) channel (right). The LyTR puncta were analyzed by spot detection in Imaris to estimate (**b**) number of spots per region, and (**c**) mean volume (µm^3^), of control and *ALiX*-RNAi virgin and mated females at 24 h post-mating. The box plots represent maximum, median and minimum values with outliers of 15–20 spermathecae per condition (From 10–15 flies); letters denote significant differences (one-way ANOVA, with multiple comparison post-hoc test, *p* < 0.05) (see also Fig. 3c). Scale bar = 10 µm.

Since interference with *ALiX*, *Hrs*, *Rab11* and *Rab7* clearly affected female fecundity, we next tested whether this disruption affects the SSC trafficking system. This was done by co-expressing CD63-GFP with *ALiX*-, *Hrs*-, *Rab11*- and *Rab7*-RNAi in the SSC and analyzing intracellular CD63-GFP levels post-mating. Silencing *Rab11* and *ALiX* significantly lowered, and *Hrs* and *Rab7* significantly elevated the number of CD63-GFP particles, while having no effect on mean particle volume at 24 h post-mating (*p* < 0.05; Fig. 6c and Supplementary Fig. 7). At 72 h post-mating, silencing of *ALiX* further reduced CD63-GFP particles (*p* < 0.05; Fig. 6d), suggesting a shift in CD63-GFP trafficking, probably for degradation^40^. Similarly, RNAi targeting of different endosomal sorting complexes required for transport (ESCRT) and ESCRT-associated proteins in HeLa cells and Drosophila, has shown that depletion of Hrs reduces the secretion of exosomes^16,34,40^. Knockdown of *ALiX* reduces exosome secretion in male Drosophila accessory gland secondary cells^16^ and alters exosomes in HeLa and human dendritic cells, changing the proportion of surface markers of CD63 exosomes and their size, with an increased proportion of medium (50–100 nm) and large (100–200 nm) exosomes^40^. To further confirm the impact of trafficking network impairment on the release of EVs, we silenced Rab7 in the SSC and used our ex vivo system to quantify the released EVs. We found a significant decrease in particle number (control:1.09 × 10^9^ ± 1.04 × 10^8^ vs. *Rab7*-RNAi: 7.22 × 10^8^ ± 1.04 × 10^8^; *p* < 0.02) and mean diameter (control: 109 nm ± 3.6 vs. *Rab7*-RNAi: 97 nm ± 3.6; *p* < 0.03), and a change in size distribution compared to controls (control: 60–120 nm vs. Rab7-RNAi: 60–90 nm) (Fig. 6e–g). In addition, *ALiX*-RNAi altered endosome maturation in the SSC; the number of Lysotracker-positive spots increased compared to control SSC and were of smaller volume (*p* < 0.05; Fig. 7), indicating an ALiX-mediated shift in endosome trafficking. These results are consistent with a reduction in the number of CD63-GFP particles in *ALiX*-RNAi SSC and suggest that silencing of *ALiX*, *Hrs*, *Rab11* and *Rab7* modifies fecundity by affecting intracellular CD63-GFP content and endosome maturation, resulting in changes in SSC secretory/endocytic activity and reduced EV release from the SSC.

### Sperm and male accessory gland proteins are essential for SSC trafficking activity

**Fig. 8:**
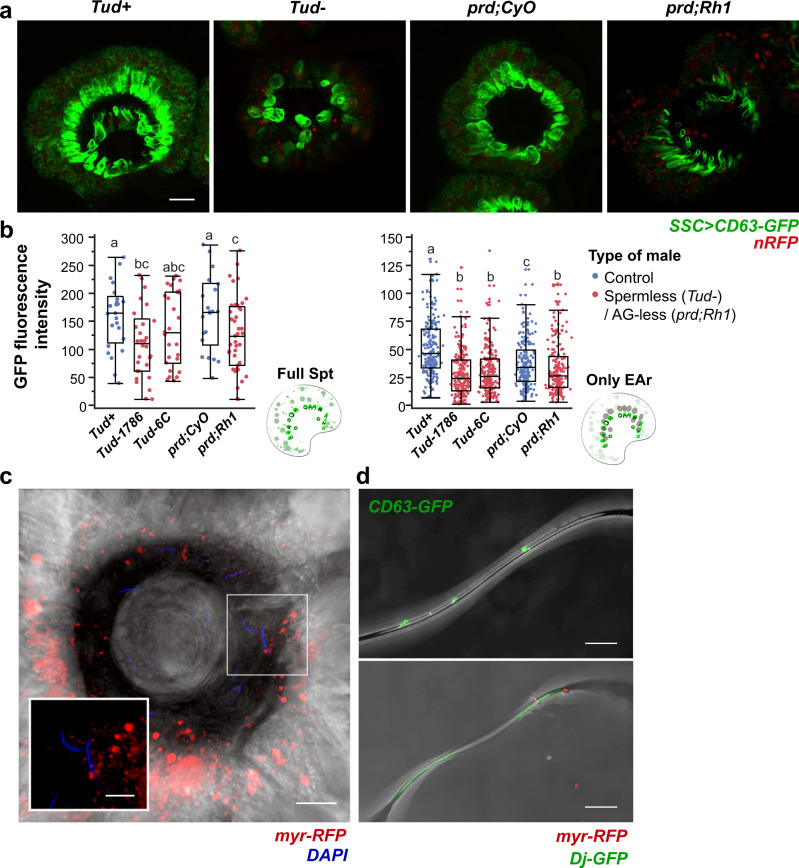
SSC secretory activity requires both sperm and male accessory gland proteins. **a** Representative confocal maximum projection images of spermathecae at 24 h post-mating expressing CD63-GFP in the SSC of females mated to the following males: control *tudor* (*Tud*^+^), *sons of tudor* (*Tud*^-^), control *prd* (*prd;CyO*), and *prd* (*prd;Rh1*). **b** Mean CD63-GFP fluorescence intensity of the whole spermatheca, and individual end apparatus reservoir (EAr). The box plot represent maximum, median and minimum values with outliers of 20 spermathecae per condition (From 11–15 flies); letters denote significant differences among treatments and were estimated by two-way ANOVA, Tukey HSD, multiple comparison post-hoc test, *p* < 0.05. **c** Micrograph of spermatheca with SSC expressing myr-RFP overlaid on the brightfield channel depicting DAPI nuclei of sperm in contact with myr-RFP vesicle-like particles in the spermatheca lumen (Supplementary Movie 6 for a 3D overview of **c** showing sperm surrounded by myr-RFP vesicle-like particles). **d** Lightning confocal micrographs of sperm isolated from spermathecae expressing CD63-GFP or myr-RFP in the SSC. Scale bar of **a**, **c** and **d** = 20 µm; inset of **c** = 5 µm.

Many of the changes occurring in females following mating, including enhanced egg-laying, are induced by seminal fluid proteins (SFPs) from the male-accessory glands. The magnitude of female response to mating depends on the quantity and composition of SFPs received via the ejaculate^1^. To determine which male-supplied components affect the SSC secretory response, we mated females expressing CD63-GFP in the SSC with males lacking sperm or accessory glands (Fig. 8). At 24 h post-mating, CD63-GFP fluorescence intensity in the whole SSC and end apparatus reservoirs decreased significantly in females mated with spermless males, compared to females mated with control males (*p* < 0.05; Fig. 8a, b). Interestingly, there was also significantly less CD63-GFP fluorescence in the SSC of females mated with males lacking accessory glands (*p* < 0.05; Fig. 8a and b). In both cases, the CD63-GFP localization in the SSC cytoplasm and end apparatus reservoirs resembled that of a virgin fly (Fig. 3a and b). Thus, sperm and accessory gland proteins are essential modulatory inputs for SSC trafficking activity at 24 h post-mating. Sperm and accessory gland proteins have also been found to regulate gene expression in the whole female^51^. Strikingly, a scan through multiple focal planes of SSC expressing myr-RFP revealed that sperm in the spermatheca lumen are immersed in myr-RFP-positive puncta (Fig. 8c, Supplementary Movie 5). Moreover, the tails of sperm isolated from spermathecae of CD63-GFP or myr-RFP SSC bear vesicle-like puncta that seems to be located in specific regions along the sperm (Fig. 8d).

Recent in-vivo and co-incubation studies have shown that male- and female-RTs-derived EVs of various organisms interact with sperm to enhance its development, functionality and survival. EVs have been observed docking on the sperm head and/or tail, being taken up by sperm, and transferring their cargo to sperm^4,16,52–57^. In Drosophila, Corrigan et al. showed that sperm transferred to the female during copulation carries male-accessory gland-derived CD63-GFP exosomes^16^. Our findings that accessory gland proteins and sperm are essential for the mediation of SSC secretory/endocytic activity may also suggest that part of the sperm effect is due to the male-accessory gland-derived EVs that are delivered to SSC via sperm. In addition, SSC-derived EVs that are secreted to the spermatheca lumen and found on the tails of stored sperm may be involved in sperm maintenance during storage.

## Conclusions

Our findings provide the first evidence that mating can modulate the secretory/endocytic activity of the SSC, and that sperm and accessory-gland proteins are essential for such mating-mediated SSC activity. We also found that in response to mating the SSC release a heterogeneous population of EVs which is enriched with exosomes and contains particles in the size-range of microvesicles. Moreover, our data indicate that these mating-induced changes in SSC trafficking alter the secretory output (e.g. release of EVs from the SSC) necessary to initiate and regulate egg-laying. We hypothesize that mating also changes the type of EVs released, and the cargo these vesicles carry. In addition, the profiles of Rab localization and CD63-GFP endosomes may indicate that the SSC have the potential to release their contents apically to the lumen towards the sperm and other regions of the female RT. We suggest that the immediate release of SSC-derived EVs post-mating (Fig. 3a, b 0 h) contributes to the terminal differentiation of the female-RT, regulated by mating^33^. This in turn leads to a functional female-RT able to support sperm storage and egg-laying. As EVs cross various biological barriers, including tissue barriers, and are targeted to discrete local and/or distant sites^58^, they may play a role, at later times post-mating (>24 h post-mating, high reproductive state), in recruiting neurons that regulate egg laying. One possible example is activating of female-specific oviposition descending neurons (oviDNs) that Wang et al. showed is needed and sufficient for egg laying^59^. SSC secretory output, including release of EVs may also induce or inhibit release of neuromodulators in the female-RT or activate other neurons outside the female-RT that are involved in the egg laying process^60,61^. Also, seminal proteins and sperm have been shown to mediate changes in neuromodulator distribution (i.e. octopamine, serotonin, dromyosuppressin) in the female-RT post-mating^60,62^. Seminal proteins and sperm could potentially regulate SSC-EV trafficking and/or release; secretion of mating-responsive SSC-derived EVs from the spermatheca could lead to changes in neuromodulators at nerve terminals along the female-RT that are involved in the egg-laying process. Alternatively, sperm and seminal proteins may regulate release in the female-RT of neuromodulators that modulate SSC secretory/endocytic activity, which are in turn involved in regulation of egg laying. Furthermore, when the central nervous system engages the muscles associated with egg laying by releasing glutamate, octopamine and/or other neurohormones at the neuromuscular junction, mating-responsive SSC-derived EVs may mediate such engaging of the muscles, thereby regulating egg laying.

The study also sheds light on SSC-female-RT routes of communication (e.g. uterus, seminal receptacle) and shows that sperm are immersed in and carries EVs released from the SSC to the spermatheca lumen. The fact that we observed SSC-derived EVs docking on sperm points to a potential role in maintaining sperm in storage and perhaps points to involvement in post-ejaculatory modifications to sperm (PEMS)^63^. For example, PEMS, promote capacitation, a widespread phenomenon across the animal kingdom that makes sperm competent to fertilize an oocyte, extends sperm longevity, or even supplies sperm with components for the oocyte at fertilization and during the first stages of zygote development^4,63,64^. The role of EVs in sperm capacitation/activation is further supported by the findings of Fereshteh et al. that in mice, sperm acquire factors from the vaginal environment which induce capacitation, and by Franchi et al. whose results demonstrated that bovine oviduct fluid EVs from the isthmus and ampulla region participate in the induction of acrosome reaction and in signaling events associated with sperm capacitation^53,54^. Since the spermatheca is able to receive and transport sperm and maintain a microenvironment that supports long-term sperm viability, it is likely that tightly regulated communication between the SSC and sperm, partially mediated by EVs, is involved in providing nutritional factors, oxygen and other necessary elements. Future studies will determine whether SSC-derived EVs act alone or in cooperation with male RT-derived EVs, and whether other female-RT- derived EVs modulate sperm maintenance and PEMS.

Finally, the involvement of EVs in SSC-female-RT routes of communication has added another layer of complexity to the process driving the switch towards a functional female-RT, and to high fertility. The precise mechanism by which male-derived signals, including EVs, affect SSC-derived trafficking has yet to be resolved. Deciphering male-female communication via EVs in Drosophila and other organisms will contribute greatly to our understanding of the different combinations of input modalities and output networks leading to high fertility.

## Methods

### Gene expression analyses

#### Datasets used

To determine gene expression enrichment related to extracellular vesicle (EVs) secretion, we used the following datasets describing gene expression in adult female tissues (1) Gene expression in the spermatheca of virgin and mated females at 3 and 6 h post-mating (microarray) reported by Prokupek et al.^21^, (2) Gene expression in female reproductive tract and whole female (RNA sequencing) reported by McDonough-Goldstein et al.^22,25^, (3) Spermatheca and whole female gene expression (microarray) reported in the FlyAtlas1 project^23^, and (4) Spermatheca and whole female gene expression (RNAseq) reported by the FlyAtlas2 project^24^. Analysis of microarray mRNA datasets was conducted in R 4.0.3 utilizing Affy package v1.68.0^65^. Probe mapping was completed using custom CDF from the Brain Array Lab^66^ (“ENSG” version 25). Probe sets labeled absent by MAS5 in the majority of spermatheca samples were excluded, and fold changes between conditions were assessed using limma 3.46.0^67^ (*p* < 0.05). RNAseq summarized read counts were obtained from NCBI GEO and FlyAtals2 web page and analyzed in R using Deseq2 1.30.1^68^. Transcripts with average read counts among analyzed samples below 1 were excluded.

#### Gene expression plots and heatmaps

GO terms (biological process) were chosen by their relevance to EV secretion and annotated gene count (>20). Expression of genes associated with particular GO terms was compared between pairs of conditions (i.e. spermatheca vs. whole body, spermatheca of virgin vs. mated at 3 or 6 h post-mating) using Kolmogorov–Smirnov test, and visualized by density plots using R. Gene expression heatmaps were generated in Excel and the expression mean-subtracted in log2 scale. Genes and samples were ordered according to hierarchical clustering, generated using Pearson correlation using Ward’s method in R.

### Drosophila strains and husbandry

The line Send1-GAL4 was provided by Mark Siegal^14^. The reporter lines for UAS-CD63-GFP^69^ with or without tub-GAL80ts, UAS-myr-mRFP w[1118]; P{w[+mC]=UAS-myr-mRFP}1^70^, UAS-sGFP^20^, and the RNAi lines for *Rab11* (y [1] v[1]; P{y [+t 7.7] v[+t1.8] = TRiP.JF02812} attp2 and P{attP,y+,w3'}VIE-260B), ALiX (w1118; P{GD7853}v32047 and y[1]sc[*]v[1];P{y[+t7.7] v[+t1.8]=TRiP.HMS00298}attP2), Hrs (y [1] SC [*] v [1]; P {y [+t7.7] v [+t1.8] = Trip.HMS00840} attP2 and y[1] sc[*] v[1]; P{y[+t7.7] v[+t1.8]=TRiP.HMS00841}attP2), and *Rab7*-RNAi (y[1] v[1]; P{y[+t7.7] v[+t1.8]=TRiP.JF02377}attP2) were provided by C. Wilson^16^. The YFP-tagged Rab11, Rab7, Rab5 and Rab35^26^ were provided by R. Maeda^71^. The lines Tudor 6C, Tudor BL:1786, prd-GAL4 and UAS-Rh1/CyO were provided by M. Wolfner. Flies were raised on standard yeast/ cornmeal/sucrose media at 25 °C and a 12:12 h light/dark cycle. All experiments were conducted on flies that were 3-day-old post-eclosion.

#### Fly husbandry

Flies were raised on a standard yeast/cornmeal/sucrose medium at 25 °C with a 12:12 h light/dark cycle. Virgin females and males of each line were collected separately and aged for 3 days. Females were mated individually with their male siblings (1:2) or kept virgin. In all cases male and female pairs were observed to record mating initiation and termination times. After copulation (of at least 10 min), virgin and mated females were kept singly in vials with fresh food at 25 ± 2 °C until examined. All experiments were conducted on 3-day-old adult flies.

### Examination of SSC secretory activity

Because the spermatheca lumen is extremely small and inaccessible, SSC secretory activity was evaluated using in-vivo visualization tools based on fluorescent reporters.

#### Evaluation of SSC secretory activity

To assess the effect of mating on the secretory activity of the SSC, we used *Send1-GAL4*, a specific driver of the SSC expressed exclusively post-eclosion^14^, to express: 1. a secretory form of GFP (UAS-sGFP). sGFP is composed of sex-peptide secretion peptide fused to GFP^20^; 2. CD63-GFP (UAS-CD63-GFP), GFP-tagged CD63 tetraspanin which is a mammalian transmembrane EV marker, used previously to mark fly EVs^16,34,69^; 3. myristoylated RFP (UAS-myr-mRFP), a post-translational modification that anchors the reporter protein to membranes of EVs^36,70^. We then visualized the route of SSC-derived secretion following sGFP, and the expression of CD63-GFP and myr-mRFP in the SSC of virgin and mated females.

#### Rab GTPases expression and localization in the SSC

Rab GTPases are associated with theendocytic and secretion pathways^72,73^. To examine the role of Rab GTPases in SSC activity, we first used YFP-tagged Rab GTPases (Yrab) fly lines: Yrab5, Yrab7, Yrab11 and Yrab35^26^ and visualized their expression and subcellular localization in virgin and mated females 24 h post-mating.

#### Pulse–chase

To assess whether mating modulate trafficking of CD63-GFP through the secretory and endosomal systems, we used a pulse–chase approach. *Send1* > *CD63-GFP*^*ts*^ virgin females were crossed with *UAS-CD63-GFP*^*ts*^ males (W; UAS-CD63-GFP, tub-Gal80ts; dsx-Gal4/SM5-TM6) and their progeny maintained at 18 °C for 3 days after eclosion. Thereafter, all the flies were pulsed for 6 h at 28 °C to repress GAL80 and induce CD63-GFP expression. We chased the cellular localization of CD63-GFP at different time points during a total of 66 h at 18 °C. To dissect the effect of mating on the CD63-GFP dynamics, 18 h into the chase, a group of flies were mated, dissected and imaged at 0, 6, 24 and 72 h post-mating, while another group was used as virgin controls for each time point. Note that after the 6 h of pulse the remaining GFP mRNAs are further transcribed^16^.

#### Effect of gene silencing on the intracellular pool of CD63-GFP

To examine whether silencing of *ALiX*, *Rab7*, *Rab11* or *Hrs* affects the intracellular pool of CD63-GFP, we co-expressed *Send1* > *CD63-GFP* with each of the selected genes (*Send1* > *CD63-GFP* > X-RNAi: *ALiX, Rab11, Hrs, Rab7*). We then imaged virgin and mated females. Mated females were imaged at 1.5, 24 and 72 h post-mating for *ALiX-RNAi* and at 24 h post-mating for *Rab11-*, *Hrs-* and *Rab7-RNAi* flies (see additional imaging details in the imaging section).

#### Evaluation of the effect of male reproductive components on the SSC activity

To determine the role that sperm or male-accessory gland proteins have on the activation of the SSC after mating, we used spermless or accessory gland-less males. Sons of Tudor spermless males were generated by crossing straight winged Tudor females with CantonS males. Males with no accessory glands were generated by crossing Prd-GAL4 with UAS-Rh1/CyO. Females expressing CD63-GFP in the SSC were mated with males lacking either sperm or accessory gland proteins, and with their fertile siblings as control. The pattern of CD63-GFP expression in the spermatheca was evaluated in virgin and 24 h post-mating females.

#### LysoTracker staining

RTs were dissected in cold Schneider’s media. The dissected tissues were incubated in Schneider’s media with 5 µM LysoTracker™ Deep Red (LyTR; Thermo Fisher Scientific, Cat. No. L12492) for 5 min. The RTs were then rinsed in fresh Schneider’s media and mounted as detailed above and imaged thereafter.

#### Dissections

The female-RT of virgin and mated flies were dissected in cold Schneider’s Drosophila media (Biological Industries). The lower RTs were isolated, cleaned of cuticle and fat-bodies and rinsed twice to remove debris. The cleaned tract was mounted on a slide in Schneider’s media (Fig. 3b–d). For the *in-tissue* CD63-GFP particle analysis assay, the tracts were mounted in antifade consisting of 70% glycerol and 2% N-propyl gallate and (Supplementary Fig. 7). The slide was sealed with a #0 coverslip and viewed immediately.

### Confocal microscopy and image processing

Spermathecae and RTs were imaged in a Leica TCS SP8 multiphoton (MP) laser scanning confocal microscope operated by the LAS X software. Fluorescence was detected by using argon excitation lasers of 488 nm (for GFP and YFP), which signal was captured by a Hybrid detector (HyD), 552 nm laser (for nRFP) captured by a conventional photomultiplier (PMT) and 638 nm (for LyTR) by a second HyD. The size and number of optical sections from different focal planes were collected according to the following experimental sets.

#### Localization of SSC-derived sGFP

RTs of females expressing sGFP specifically in the SSC were imaged with the 63x/1.2 objective, 0.75x digital zoom and 1024 × 1024 lines using the tile scan experiment. The tiles (stacks of an average of 20 optical sections) were subsequently automatically stitched together to create a full image of the whole RT.

#### Expression of Rab proteins in the spermatheca

Spermathecae of the different YRab lines (see above) were imaged to characterize their expression and spatial distribution in the SSC. The images were captured using the 63x/1.2 objective, digital zoom of 1.5 or 3.6x with HyD on standard mode, gain of 70 and a resolution of 1024 × 1024 lines; each frame was averaged three times to eliminate false-positive signal, and an average of 30 optical sections of 0.5 µM per organ. To analyze the localization patterns of the YRabs in the different subcellular regions (apical, medial or basal), we created a template of eight radial sections (“pie chart style”) and laid it over the maximum projection images of the glands (one section always contained the stalk of the spermatheca, hence was not analyzed; Supplemental Fig. 2b). Each radial section was subdivided into apical, medial or basal subcellular regions, according to the proximity to the lumen of the spermatheca. We followed Dunst et al.^14^ terminology to describe the appearance and analyze the intracellular distribution of the different YRab proteins in the SSC. YRabs expressed in the SSC are either associated with large membranal compartments (LMC), small intracellular membranal punctate (punctate), or with non-membrane-associated pool or small vesicles below the resolution of the light microscope (diffuse). Because each YRab can exist in different pools within the cell (LMC, punctate, diffuse), we examined whether the YRabs expressed / not expressed in each subcellular localization, for each of the pools, in every radial section of virgin and mated SSC (Fig. 2a, c). In addition, we evaluated if each of the YRabs is enriched in a specific subcellular region, for each of the pools, in every radial section of virgin and mated SSC (Supplementary Fig. 2c). The abundance of the YRab was evaluated as low (1) or high (2) for punctate and LMC pools, and present (1) or absent (0) for diffuse pool. Since the expression of the YRabs was not consistent over the glands and there were regions in which no YRab was expressed (in contrast to the expression of other markers like CD63-GFP that were found expressed in all SSC), the analysis of the YRab expression was performed only on the expressing regions to reflect the status of active cells. Although the end apparatus reservoirs are found within the apical subcellular region, the reservoirs were given special attention as they are independent membranous structures. The end apparatuses might inform which of the YRabs is necessary for release of secretions to the spermatheca lumen in virgin and mated females and report on the potential secretory activity of the spermatheca.

#### Imaging the SSC secretory activity

We evaluated SSC secretory activity by using different fluorescent reporters (*+/UAS-CD63-GFP; +/Send1-nRFP, +/UAS-myr-mRFP; +/Send1-nRFP*) and LyTR. To assess the GFP fluorescence intensity level, or characterize RFP expression in the SSC, an average of 80 optical sections per spermatheca each of 0.5 µM (the size of the spermatheca changes post-mating) were captured using the HyD with a gain of 50 or 100 (for CD63-GFP and myr-RFP respectively), a resolution of 1024 × 1024 lines, averaging each frame 3 times. LyTR was captured with a PMT at 770.

#### Lightning imaging of the spermatheca, sperm and CD63-GFP EVs

The spermatheca in Fig. 3e was imaged using lightning mode of the Leica LAS X. The image was captured with a resolution of 2472 × 2472 lines, averaging each line twice, 76 optical sections of 0.5 µM and with a zoom of 1.28x. The sperm in Fig. 8d was first dissected out from spermatheca expressing CD63-GFP or myr-RFP in the SSC. The sperm was imaged capturing 91 sections of 0.1 µM using lightning mode at a resolution of 744 × 744 lines, averaging each frame and line twice and a digital zoom of 4.5x. The CD63-GFP EVs in Supplemental Fig. 5a were from the spent media of 75 spermathecae. All images were automatically processed by the lightning deconvolution feature of LAS X.

#### Live imaging of the spermatheca activity

The female-RTs were dissected as stated above, and the spermatheca was sectioned from the tract. The spermathecae were placed on a coverslip in Schneider’s media, dried briefly to allow it to lay on the surface and immediately covered by a drop of fibrin in Schneider’s media (1:3). Thereafter, one part of thrombin was added to the fibrin and the mix was allowed to clot for 3 min before adding 50 µl of Schneider’s at 25 °C. SSC expressing CD63-GFP were imaged using a 488 nm laser at 1.6%, captured by a HyD at 60% for GFP, and a 552 nm laser at 1%, captured by a PMT at 770V for nRFP and digital zoom of 1 for whole spermatheca or 2.25 for zoom to the SSC.

#### Imaging CD63-GFP particles in the SSC

To analyze CD63-GFP intracellular pool in the SSC, we focused and zoomed to a specific section of the spermatheca. The images were captured using a digital zoom of 2.5x focusing on the proximal lower quarter of the spermatheca where active SSC were observed. The imaging was performed with the 488 nm laser at 3% and captured with the HyD using photon counting mode, with a resolution of 1024 × 1024 lines, averaging frames 4 times to eliminate false-positive signal, and 50–59 optical sections of 0.5 µM per organ.

*Note that for all the performed experiments we analyzed both spermathecae to capture the full response*.

### Image analyses

#### Measurement of GFP intensity level

The analysis of CD63-GFP in the SSC was performed using Fiji^74^. The area, GFP mean fluorescence intensity and corrected integrated total fluorescence (CITF) of different SSC regions were evaluated by delimiting an ROI around the whole spermatheca, end apparatus region, cytoplasm region and lumen region (Fig. 3b; Supplementary Fig. 3b; Supplementary Fig. 4a; Fig. 8b). For individual end apparatus reservoirs, ROI of 10 end apparatus reservoirs were captured per spermatheca, for which width and length were also registered (Supplementary Fig. 3d, e and Supplementary Fig. 4c).

#### Evaluation of LysoTracker spots in the SSC

The acidic puncta stained with LysoTracker in the SSC were detected using the spots tool of Imaris 8.4 (Bitplane). The number, volume of the particles detected in the ROI analyzed and GFP mean intensity level were recorded using the vantage tool of Imaris (Fig. 3c and Fig. 7b, c).

#### Evaluation of myr-RFP fluorescence in the SSC

The spatial localization of myr-RFP in the SSC was evaluated using Imaris. To differentiate the membranal myr-RFP (magenta) from the nuclear RFP (red), the image was segmented based on the size and volume of the nuclei. An independent channel was created for these nuclei, which was subtracted from the total RFP channel to receive a unique channel consisting only the myr-RFP (shown in magenta, Fig. 3d, f). Whole spermathecae of virgin or 24 h post-mating females were observed in 3D; an ortho slicer was adjusted manually in order to scroll through the focal planes to visualize and quantify the number of myr-RFP-positive EArs (Fig. 3d).

#### Determining SSC activity and endosome localization in the pulse–chase assay

We examined CD63-GFP expression and spatial localization in the SSC at different chase times of virgin and mated females. The SSC were marked as “active” only if their end apparatus reservoirs were CD63-GFP-positive (Fig. 4c, d). The “active” SSC were then classified based on the intracellular location of endosomes at their apical, medial or basal regions (Fig. 4e) or a combination of the locations as shown in the schematic of Supplementary Fig. 4b.

#### Analysis of CD63-GFP particles in the SSC

CD63-GFP puncta were examined in the SSC using the surface tool of Imaris. For this, a constant ROI of x:719, y:677 and z:56 pixels in size was created and applied equally to all the spermathecae measured. GFP was selected as source channel, from which the smooth feature was applied to a surface detail of 0.1 µM and the background subtracted to a diameter of 0.9 µM. The threshold of the background was adjusted between 1.3 and 12.2. Finally, the surfaces were filtered by volume applying an upper limit of 1 µm^3^ and a lower of 1.4 × 10^−5^ µm^3^. The volume of the particles registered in the ROI and mean GFP were acquired using the vantage tool (Fig. 6c, d).

### Ex vivo spermatheca culture

To evaluate and characterize the release of EVs from the spermatheca, we developed an ex vivo culture system to collect spermatheca derived EVs. Spermathecae were separated from dissected female-RTs in Schneider’s Drosophila media (Biological Industries, Cat. No. 01-15-1A), as stated above, and rinsed three times in clean Schneider’s media. A group of 25 spermathecae were then transferred to a drop of 10 µl of 0.2 µm filtered Schneider’s media alone or with 10 µM 20-hydroxyecdysone (Sigma, Cat. No. H5142), on a slide maintained in a humid chamber at 25 °C. After 2 h of incubation, the spermathecae and spent media were transferred to a 0.5ml Eppendorf tube. The well was then rinsed with 10 µl of 0.2 µm filtered Schneider’s media and transferred to the same Eppendorf tube. We added ecdysone to the media because the concentration of EVs secreted to the media without supplement of ecdysone was at the threshold of accuracy for the tools available for nanoparticle analysis. Ecdysone significantly enhanced the release of EVs from the spermatheca but did not change the size range of EV population or the mean EV diameter (Fig. 5g–i, *P* < 0.05; Supplementary movie 5). The effect of ecdysone as promoter of secretory activity has been observed in the salivary glands of Drosophila larvae^75^.

### Evaluation of EVs produced by the spermatheca in the ex vivo culture

All the aliquots of frozen isolated spent media were allowed to thaw on ice before any characterization step.

#### EV isolation and purification

Spermatheca from SSC expressing Rab7-RNAi or control, SSC expressing CD63-GFP or CantonS flies were cultured ex vivo as described. The collected spent media was centrifuged at 2000 × g (Eppendorf 5424R, Rotor FA-45-24-11) for 10 min and the supernatant was transferred to a clean 0.5ml tube and centrifuged at 13,000 × g for 10 min. The final supernatant was transferred to a clean 0.5ml tube and stored at −80 °C.

#### AcouSort isolation of EVs

Extracellular vesicles from 200 spermathecae were collected as above and then isolated by acoustic sorting using the AcouTrap system (AcouSort AB). We used the AcouTrap isolated EVs for characterization by AFM (Fig. 5f, Supplemental Fig. 5d–g).

#### Nanoparticle Tracking Analysis (NTA) by NanoSight

Spent media of 3 different collections were pooled together to complete a sample of 150 spermathecae. Thereafter, the samples were diluted in 1ml of PBS and loaded in the sample chamber of the NanoSight NS300 (Malvern Panalytical). The NTA was performed using the 405nm laser, in three recordings of 1 min each, using a camera level of 13 and gain of 5 and loading 100µl per read. The analysis was performed using a threshold of 5 and gain of 15 (Fig. 6e–g).

#### Characterization using the Nanoimager: Single-particle tracking and dSTORM

##### Coverslip preparation

1.5H high precision coverslips (Marinfeld, Cat. No. 0101142) were cleaned by sonication for 5 min in acetone followed by a brief rinse in deionized water. Subsequently coverslips were sonicated in 1M KOH for 30 min then rinsed with deionized water. The remaining liquid from the surface of the coverslip was removed using high pressure N_2_. The slides were coated with poly-L-Lysine for 2 h at 37 °C. To minimize non-specific binding of EVs to the surface of the coverslip, blocking was performed for 30 min at room temperature with a 10% BSA (VWR Chemicals, Cat. No. 422351S) solution in PBS (0.1M, pH7.4, Sigma-Aldrich, Cat. No. P4417-100TAB). Finally, the blocking solution was aspirated from the coverslip, and the excess BSA was removed by three consecutive washes of PBS (1 min each). The coverslips were immediately used for tracking experiments or for super resolution imaging to avoid drying of the surface.

#### Size evaluation by single-particle tracking

##### EV membrane labeling

Spent media from 25 spermathecae (from CantonS flies) in Schneider’s alone or 10 µM ecdysone were stained with 2.5ng/µl CellMask Orange plasma membrane dye (Invitrogen, Cat. No. C10045) for 10 min at 37 °C. The labeled EVs were immediately added to the coverslip wells prior to single-particle measurements. The spent media of SSC expressing CD63-GFP was not labeled.

##### Single-particle-tracking analysis

Measurements were acquired on the Nanoimager S Mark II from ONI (Oxford Nanoimaging) equipped with 405 nm/150 mW, 473 nm/1W, 532 nm/1W, 640 nm/1W lasers and dual emission channels split at 640 nm. 1000 frames were acquired at 100 Hz (*n* = 4 per condition) for GFP (no CellMask) and CellMask with 10% laser power for the 473 and 532 nm lasers respectively in highly inclined laminated optical sheet (HILO) mode. Data was processed in NimOS (Version 1.7.1.10213) from ONI (Fig. 5a, g–i).

#### Imaging CD63-GFP EVs by Super Resolution Microscopy

##### EV immunostaining

EVs isolated from SSC expressing CD63-GFP (*n* = 25 spermathecae) were allowed to bind overnight at 4 °C to the surface of poly-l-lysine coated high precision coverslips passivated as previously mentioned. EV solution was gently removed from the wells and replaced with 10 % BSA solution in PBS. After a 30 min blocking step Alexa Fluor 555 labeled (Invitrogen, Cat. No. A10470) anti-CD63 antibody (Abcam, Cat. No. ab193349) was added directly to the blocking solution and incubated overnight at 4 °C. Prior to imaging EVs were washed with PBS three times for 1 min.

##### dSTORM imaging of coverslip bound EVs

PBS was exchanged for ONI B-cubed imaging buffer and dSTORM images were acquired using the Nanoimager S Mark II. As GFP is a non-photoswitchable dye, only 500 frames were acquired using the 473nm laser followed by acquisition of 5000 frames using the 532nm laser for the single-molecule localization of the Alexa Fluor 555 labeled antibodies against CD63 at 30 Hz. In order to achieve optimal signal to noise ratio, single-molecule data was acquired in total internal reflection fluorescence (TIRF) mode.

##### Single molecule-localization filtering

Filtering was performed in NimOS from ONI. Alexa Fluor 555 localization were filtered based on photon count, localization precision and point spread function shape in the *x*/*y* axis. As GFP is unable to photoswitch, single-molecule localization filtering does not apply for data acquired in the green channel (Fig. 5c).

#### Detection of CD63 in the particles by ExoView

To confirm the presence of CD63 positive vesicles in the spent media we used ExoView. Spent media from 100 spermathecae that expressed CD63-GFP in the SSC was analyzed by ExoView using the ExoView Tetraspanin Plasma Kit (NanoView Biosciences, USA). Samples were diluted 1:3 in manufacturer supplied buffer, solution A, and incubated overnight at room temperature on ExoView Plasma Chips. Chips were washed three times in solution A, prior to incubation with fluorescent tetraspanin antibodies. Labeling antibodies consisted of anti-CD81 Alexa 555 and anti-CD63 Alexa 647. Antibodies were diluted 1:1200 as per manufacturer’s instructions and incubated on chips for 1 h at room temperature. Chips were then washed in kit supplied buffers, dried, and imaged by the ExoView R100 using nScan v2.8.9. Data was analyzed using NanoViewer 2.8.9. Fluorescent cut-offs were set relative to the MIgG control (Fig. 5b).

#### Scanning of EVs by Atomic Force Microscopy

A piece of 1 × 1 cm of mica (Grade V-1, SPI Chem, CAS#12001-26-2) was epoxy glued to a conventional glass slide and cleaved three times with cello-tape to obtain a homogeneous surface. The mica was functionalized with 100 µl of MgCl 10 mM for 3 min. Thereafter 5 µl of the spent media of 200 ecdysone-stimulated spermathecae isolated by acoustic sorting were transferred into the mica and incubated for 5 min, followed by the addition of 100 µl of Schneider’s for scanning. We scanned the samples using a NanoWizard4 AFM (JPK—Bruker) and a qp-BioAC-CI probe (CB 3 cantilever, Nanosensors, Cat. qp-BioAC-CI-10), in liquid. We also scanned the samples in QI mode, in which a force curve is measured on every pixel (Fig. 5f). From these force curves, we calculated the adhesion force and Young’s modulus using JPK Data Processing Software. The profiles of adhesion and Young’s modulus were created by measuring the mean value of the particle in three different areas of 5 × 5 µm (Supplementary Fig. 5d–g). The diameter (nm) was obtained by creating a cross section of each EV and measuring the base of the profile.

#### Characterization of EVs by transmission electron microscopy (TEM)

The spent media from 100 spermathecae of SSC expressing CD63-GFP were collected using the ex vivo organ culture and processed as above.

##### Negative staining

Three microliters of EV samples were loaded onto formvar-carbon-coated copper grids and after 1 min washed in two drops of DDW by touch and absorbed on filter paper. The grid was then placed on a drop of 1% Uranyl acetate for 10 s, absorbed on filter paper. Images were obtained using a Jeol7800 STEM at 30KV.

##### TEM at cryogenic temperature (Cryo-TEM)

Cryo-TEM was used to further characterize the EVs. Vitrified specimens were prepared on a copper grid coated with a perforated lacey carbon 300 mesh (Ted Pella Inc.). A 2.5 µl drop from the EV sample was applied to the grid and blotted with a filter paper to form a thin liquid film of solution. The blotted sample were immediately plunged into liquid ethane at its freezing point (−183 °C). The procedure was performed automatically in the Plunger (Leica EM GP). The samples were imaged using a FEI Talos F200C TEM, at 200kV maintained at −180 °C; and images are recorded on a FEI Ceta 16M camera (4k × 4k CMOS sensor) at low dose conditions, to minimize electron beam radiation damage. The Cryo-TEM was done at the Ilse Katz Institute for Nanoscale Science and Technology Ben-Gurion University of the Negev, Beer Sheva, Israel.

### Female reproductive success

To test if silencing of *ALiX*, *Hrs*, *Rab11* and *Rab7* has an effect on female fecundity and/or fertility, we used the *Send1-GAL4* driver to express the *UAS-RNAi* constructs of *ALiX*, *Hrs*, *Rab11* and *Rab7*. We examined two independent RNAi fly lines for each gene, beside Rab7. Female progeny that did not receive the *GAL4* driver served as controls. Virgin females expressing the RNAi or control were mated with 3-day-old virgin males from the RNAi parental line. In all the experiments male and female pairs were observed and the following parameters were recorded: (1) latency to mate: the time from introduction of the couple until copulation; (2) copulation duration: the time from mating start to finish. Females that mated <10 min were excluded from the study. After mating the males were removed and the females were kept individually and transferred to fresh food vials after 6 h and then every 24 h. The number of eggs laid by each female was counted at 6, 24, 48, 72 h and 8 days post-mating as was the number of adult progenies produced from these eggs (Fig. 6a, b and Supplemental Fig. 6).

### Statistical analysis

The analyses were performed using JMP Pro 13 or SPSS V26.0.

#### YRab localization patterns

To test if differences in localization pattern of the YRabs examined could reflect their localization in the subcellular regions of virgin versus mated spermathecae (Fig. 2c), we first used the relative risk ratio or Incident Risk Ratio (IRR) test. IRR test is based on the ODDS ratio between virgin versus mated expressed value over the total^76,77^. In this case the null hypothesis H0 was ODDS = 1, versus the alternative H1: elsewise. Confidence intervals (95%) were calculated for rejection decisions. Next, to examine if the YRab abundance would change upon mating (Supplementary Fig. 2c), we applied the two-proportion comparison test corrected by the Benjamini–Hochberg correction for multiple comparisons^78^ based on ranked *p-*values, in which maximum allowed *p* was 0.20.

#### CD63-GFP Fluorescence intensity level

The values of area and mean GFP intensity level and GFP corrected integrated total fluorescence (CITF) were first corrected to their background. The data corresponding to whole SSC and end apparatus region were normalized by dividing each one of them to their corresponding area of the spermatheca lumen (Fig. 3b, Fig. 8b and Supplementary Fig. 3b, c). Differences among times post-mating were estimated by one-way ANOVA, with nonparametric Wilcoxon multiple comparison post-hoc test (*P* < 0.05).

#### myr-RFP analysis

The number of myr-RFP-positive end apparatus reservoirs in virgin and mated females at 24 h post-mating (Fig. 3d) were quantified. The difference in number of myr-RFP-positive EArs between conditions were evaluated by one-way analysis of variances (*P* < 0.0001).

#### LysoTracker spots

The total number and mean volume of Lysotracker spots (detected in Imaris) of virgin and mated females at 24 h post-mating in the SSC of control or ALiX-RNAi females were compared (Fig. 3c and Fig. 7b, c). The effects of RNAi, time post-mating and their interaction were estimated by two-way ANOVA, followed by Tukey HSD post-hoc test (*P* < 0.05) to assess differences between the experimental groups.

#### In-tissue CD63-GFP particle analysis

To determine whether silencing of *ALiX*, *Rab11*, *Rab7* and *Hrs*, specifically in the SSC, affect the intracellular pool of SSC-CD63-GFP, we compared the count of CD63-GFP particles in each RNAi line and its corresponding no-RNAi control females (Fig. 6c, d). For that, we assessed the total number of CD63-GFP particles in the SSC per region of analysis. To measure differences in the total number and mean diameter of particles between the RNAi lines (*ALiX*-RNAi, *Rab11*-RNAi*, Rab7*-RNAi and *Hrs*-RNAi) and their corresponding controls at 24 h post-mating, we applied GLM with Poisson distribution for the number of particles followed by a post-hoc pairwise comparisons with Bonferroni correction (*P* < 0.05). To validate if the detected effect was due to the RNAi treatment and not to the amount of data collected, the particle data from the groups of SSC was resampled to 10 groups of randomly selected but equally distributed data. The analysis and significance were re-estimated as described above (GLM with Poisson distribution). We then compared the total number of CD63-GFP particles in the SSC of *ALiX-*RNAi and no-RNAi control at 1.5, 24 and 72 h post-mating using a two-way ANOVA to assay effects of RNAi, time post-mating and interactions, followed by Tukey HSD multiple comparisons post-hoc test (Fig. 6d; *P* < 0.05). Measurements correspond to the analysis of 20–37 spermathecae per condition.

#### Analysis of nanoparticle tracking

The analysis of *Rab7*-RNAi and control SSC particles (measured by NanoSight Fig. 6e–g), is the result of 3 replicates of 150 spermatheca per treatment, each pooled from 3 collections. The analysis of Ecdysone or non-stimulated SSC particles (measured by Nanoimager Fig. 5g–i) was performed for two samples of 25 spermathecae per treatment. The total number and mean size of particles released were compared by One-way analysis of variances (*P* < 0.05). In addition, we calculated a weighted mean size based on the number of particles at each size category, where size was the particle diameter ranging from 30.5 to 350.5 nm by units of 1. More formally, $Mean={\sum }_{i=1}^{320}{w}_{i}\ast {S}_{i}$, *w* = weight or the ratio between number of particles at that size over the total number of particles, *s* = diameter, and *i* = 1,2,…,320 for each measured diameter. The mean was the sum of the weighted sizes. A comparative analysis (UniANOVA) was performed to estimate the weighted mean difference between treatment (*n* = 12) and control (*n* = 12); *P* < 0.01.

#### Pulse-chase experiment

The spermathecae of pulsed virgin and mated females were sampled at different times of chase and different parameters were evaluated. The differences in the number of end apparatus with GFP (Fig. 4c), number of SSC with endosomes (Fig. 4d), the heatmap that show percentage of endosome occurrence in the apical, medial and basal regions per time point (Fig. 4e), the spermatheca GFP fluorescence intensity (Supplementary Fig. 4a), the heatmap that displays the percentage of SSC with endosomes in the designated regions per time point (Supplementary Fig. 4b), and mean end apparatus area (µm^2^) (Supplementary Fig. 4c) in virgin and mated SSC post-pulse were analyzed using two-way ANOVA, to evaluate the effect of time, mating and their interaction. Individual differences between time points were assessed with Tukey HSD multiple comparison post-hoc test (*P* < 0.05). Measurements correspond to the analysis of 10–15 spermathecae per condition.

#### Fertility and fecundity assessment

The analysis was conducted using R. Since we found females that never deposited eggs, the egg count distribution was zero-inflated using a zero-inflated model. To examine the effect of silencing *ALiX, Rab11, Rab7* and *Hrs* on fecundity, logistic regression was used. The effect of gene silencing on the egg rate deposition of the egg-laying females was analyzed by One-way ANOVA (*P* < 0.05). The onset of egg deposition is defined as the first time point at which the female laid eggs. The effect of silencing on the onset of egg deposition was analyzed by ordinal regression. In all analyses the explanatory factors were RNAi treatment, replica and interaction (Fig. 6a, b and Supplementary Fig. 6)

### Reporting summary

Further information on research design is available in the Nature Research Reporting Summary linked to this article.

## Supplementary information

Supplementary Information

Description of Additional Supplementary Files

Supplementary movie 1

Supplementary movie 2

Supplementary movie 3

Supplementary movie 4

Supplementary movie 5

Supplementary movie 6

Supplementary data 1

Supplementary data 2

Reporting Summary

## Data Availability

Raw images and source data (Supplementary Data 1 and 2) are available in the BioStudies repository (https://www.ebi.ac.uk/biostudies) under the accession number S-BIAD66.
