# Supplementary material for: Male-female communication enhances release of extracellular vesicles leading to high fertility in Drosophila
Source: Commun Biol. 2022 Aug 13;5:815. doi: 10.1038/s42003-022-03770-6 (PMC9376107; doi:10.1038/s42003-022-03770-6)
Supplement: Supplementary file 1 — Supplementary Information [file 42003_2022_3770_MOESM1_ESM.pdf]

Male-female communication enhances release of extracellular vesicles leading to high fertility in Drosophila

Javier Arturo Sanchez-Lopez[1], Shai Twena[1], Ido Apel[1], Shani Chen Kornhaeuser[1], Michael Chasnitsky[2] Andras G. Miklosi[3], Perla J. Vega-Dominguez[3], Alex Shephard[4], Amir Hefetz[5], Yael Heifetz[1*]

[1]Department of Entomology, The Hebrew University of Jerusalem, Rehovot, 76100, Israel; [2]Institute of Biochemistry, Food Science and Nutrition, The Hebrew University of Jerusalem, Rehovot, 76100, Israel; [3]ONI (Oxford Nanoimaging), Jordan Hill, Banbury Road, Oxford, OX2 8TA, United Kingdom; [4]NanoView Biosciences, Malvern Hills Science Park, Geraldine Road, Malvern, WR14 3SZ, United Kingdom; [5]DataGraph, Holon, 5880820, Israel; [*] corresponding author: yael.heifetz@mail.huji.ac.il

This PDF file includes:

Supplementary Figures S1 to S7

Other supplementary materials for this manuscript include the following:

Supplementary Movies S1 to S6

Supplementary Data 1 and 2

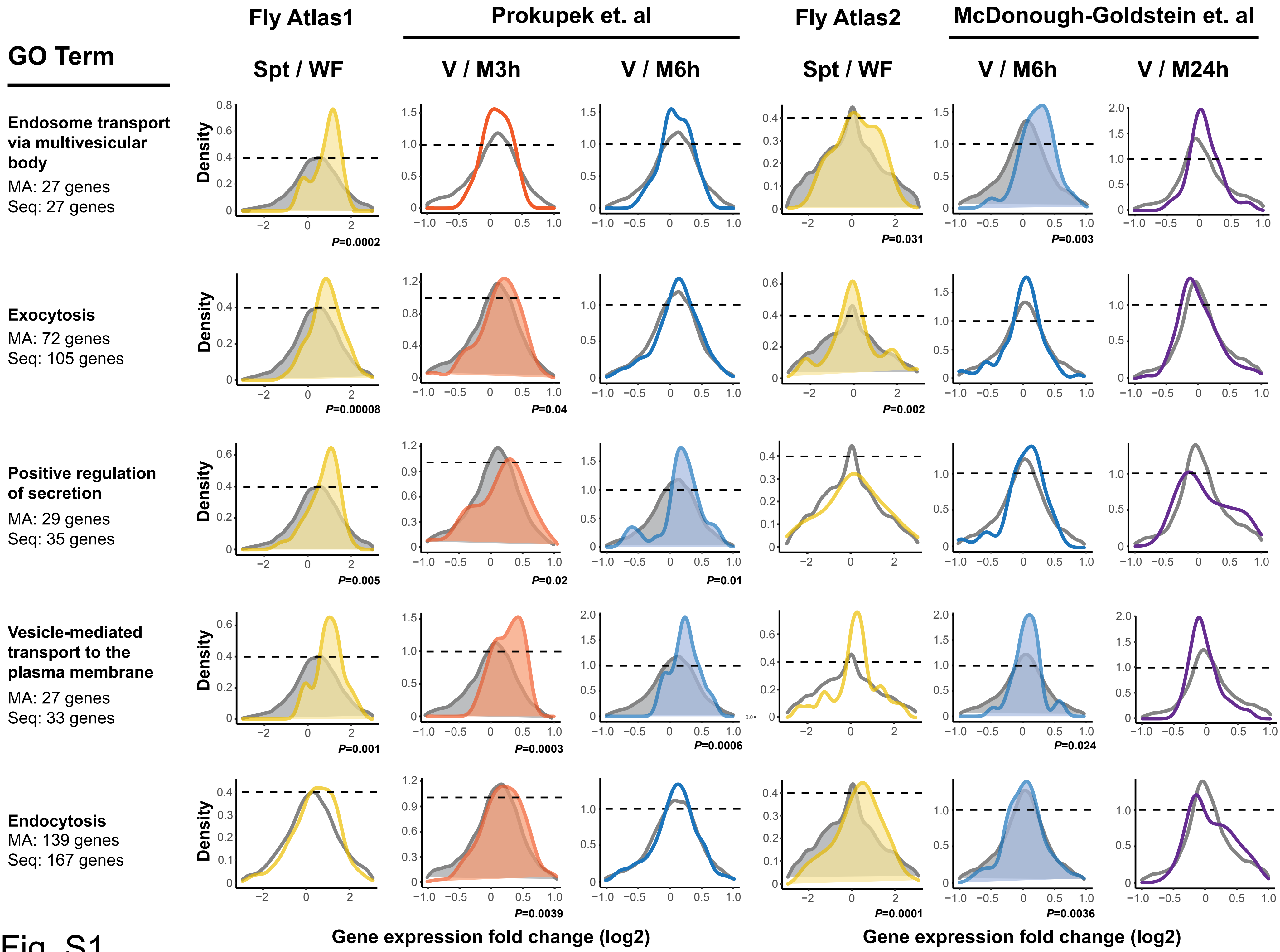
Fly Atlas1
Prokupek et. al
Fly Atlas2
McDonough-Goldstein et. al
GO Term
Spt / WF
V / M3h
V / M6h
Spt / WF
V / M6h
V / M24h
Endosome transport via multivesicular body
MA: 27 genes
Seq: 27 genes
Exocytosis
MA: 72 genes
Seq: 105 genes
Positive regulation of secretion
MA: 29 genes
Seq: 35 genes
Vesicle-mediated transport to the plasma membrane
MA: 27 genes
Seq: 33 genes
Endocytosis
MA: 139 genes
Seq: 167 genes
Density
P=0.0002
P=0.031
P=0.003
P=0.00008
P=0.04
P=0.002
P=0.005
P=0.02
P=0.01
P=0.001
P=0.0003
P=0.0006
P=0.024
P=0.0039
P=0.0001
P=0.0036
Gene expression fold change (log2)
Gene expression fold change (log2)

Fig. S1

Supplementary Fig. 1. Genes associated with GO terms related to EV exocytosis and endocytosis are enriched in the spermatheca and modulated by mating.

The presented GO terms were chosen based on their association with EV secretion and/or uptake. The density plots show the distribution of expression fold-change (log2) of genes associated with particular GO term (i.e. biological process; see Supplementary Data 1 for genes included in each term). The analysis is based on: FlyAtlas1 (expression microarray)[22], and FlyAtlas2 (RNAseq)[23] datasets for **Spt/WF** [fold change of genes expressed in virgin spermatheca (Spt) versus their expression in the whole female (WF), contrasting genes associated with the designated GO-term (yellow) against all the genes expressed in the spermatheca (gray)]; Prokupek et al datasets for **V/M3h**, **V/M6h** [fold change of genes expressed in virgin spermatheca versus 3 and 6 hours post-mating (orange and blue respectively), against all genes expressed in the spermatheca (gray)][20]; McDonough-Goldstein et al. **V/M6h**, **V/M24h** dataset for [fold change of genes expressed in virgin spermatheca versus 6 and 24 hours post-mating (blue and purple respectively), against all genes expressed in the spermatheca (gray)][21]. Broken lines highlight the same reference density for each experimental contrast. The distributions of the contrasted experimental conditions were compared by the Kolmogorov–Smirnov test; $p<0.05$, visually depicted as full colored curves (see also **Supplementary Data 1**).

Fig. S2

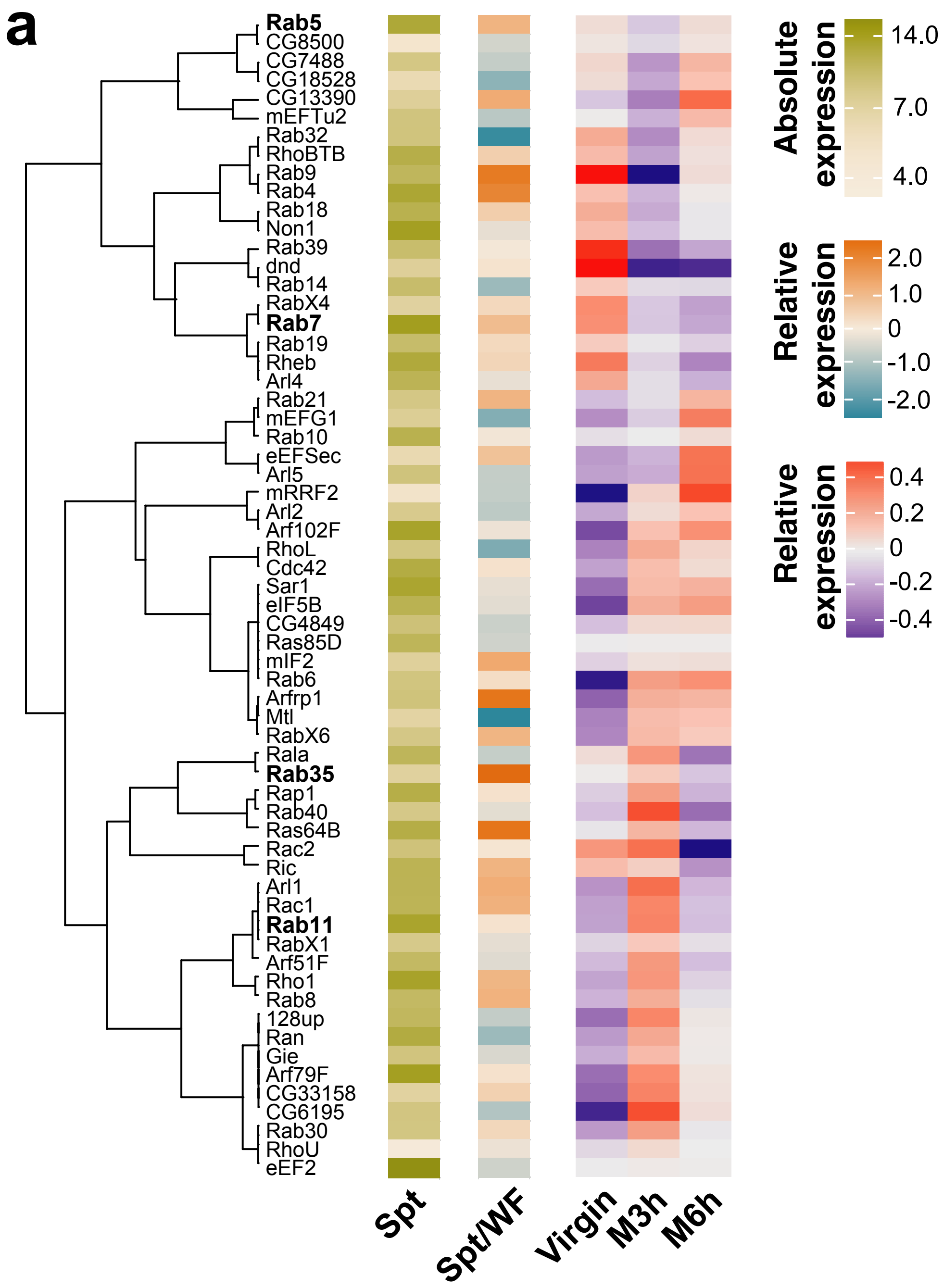
a
Rab5
CG8500
CG7488
CG18528
CG13390
mEFTu2
Rab32
RhoBTB
Rab9
Rab4
Rab18
Non1
Rab39
dnd
Rab14
RabX4
Rab7
Rab19
Rheb
Arl4
Rab21
mEFG1
Rab10
eEFSec
Arl5
mRRF2
Arl2
Arf102F
RhoL
Cdc42
Sar1
eIF5B
CG4849
Ras85D
mIF2
Rab6
Arfrp1
Mtl
RabX6
Rala
Rab35
Rap1
Rab40
Ras64B
Rac2
Ric
Arl1
Rac1
Rab11
RabX1
Arf51F
Rho1
Rab8
128up
Ran
Gie
Arf79F
CG33158
CG6195
Rab30
RhoU
eEF2
Spt
Spt/WF
Virgin
M3h
M6h
Absolute expression
14.0
7.0
4.0
Relative expression
2.0
1.0
0
-1.0
-2.0
Relative expression
0.4
0.2
0
-0.2
-0.4

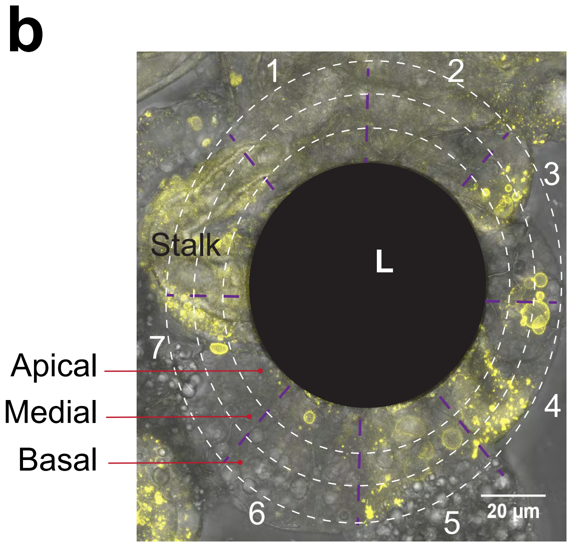
b
1
2
3
4
5
6
7
Stalk
L
Apical
Medial
Basal
20 µm

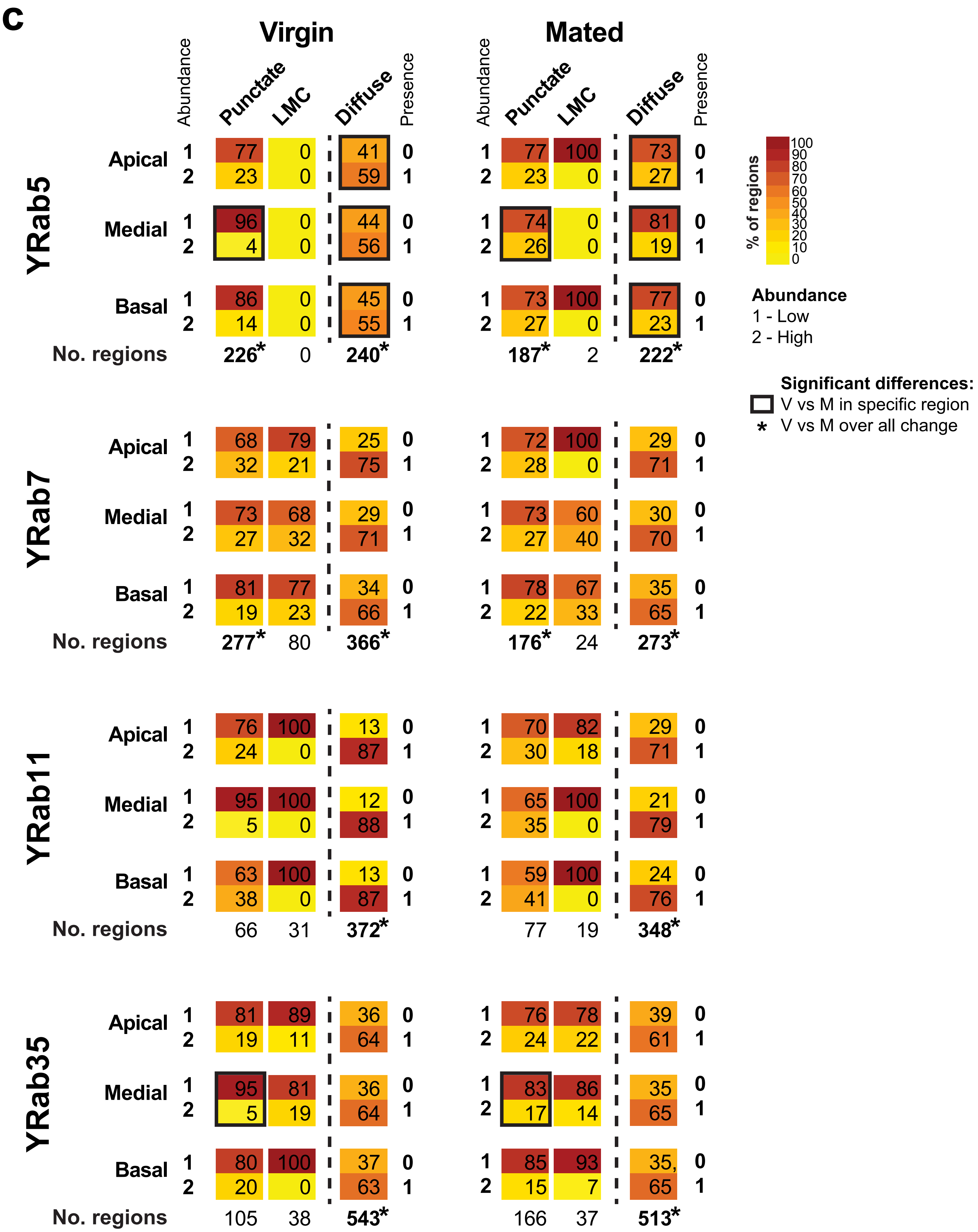
c
Virgin
Mated
Abundance
Punctate
LMC
Diffuse
Presence
YRab5
Apical 1 77 0 41 0 | 1 77 100 73 0
2 23 0 59 1 | 2 23 0 27 1
Medial 1 96 0 44 0 | 1 74 0 81 0
2 4 0 56 1 | 2 26 0 19 1
Basal 1 86 0 45 0 | 1 73 100 77 0
2 14 0 55 1 | 2 27 0 23 1
No. regions 226* 0 240* | 187* 2 222*
YRab7
Apical 1 68 79 25 0 | 1 72 100 29 0
2 32 21 75 1 | 2 28 0 71 1
Medial 1 73 68 29 0 | 1 73 60 30 0
2 27 32 71 1 | 2 27 40 70 1
Basal 1 81 77 34 0 | 1 78 67 35 0
2 19 23 66 1 | 2 22 33 65 1
No. regions 277* 80 366* | 176* 24 273*
YRab11
Apical 1 76 100 13 0 | 1 70 82 29 0
2 24 0 87 1 | 2 30 18 71 1
Medial 1 95 100 12 0 | 1 65 100 21 0
2 5 0 88 1 | 2 35 0 79 1
Basal 1 63 100 13 0 | 1 59 100 24 0
2 38 0 87 1 | 2 41 0 76 1
No. regions 66 31 372* | 77 19 348*
YRab35
Apical 1 81 89 36 0 | 1 76 78 39 0
2 19 11 64 1 | 2 24 22 61 1
Medial 1 95 81 36 0 | 1 83 86 35 0
2 5 19 64 1 | 2 17 14 65 1
Basal 1 80 100 37 0 | 1 85 93 35, 0
2 20 0 63 1 | 2 15 7 65 1
No. regions 105 38 543* | 166 37 513*
% of regions
100 90 80 70 60 50 40 30 20 10 0
Abundance
1 - Low
2 - High
Significant differences:
V vs M in specific region
* V vs M over all change

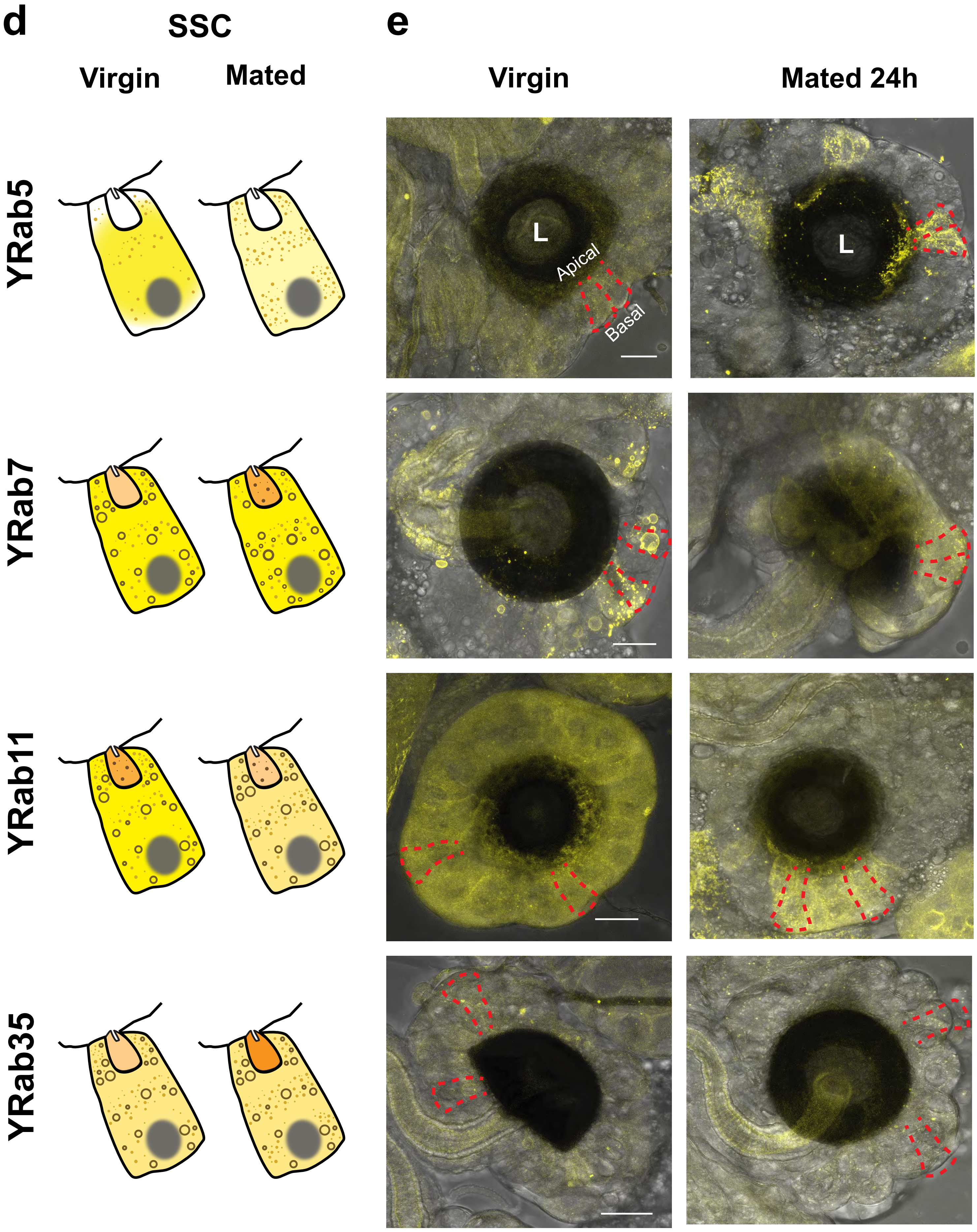
d
SSC
Virgin
Mated
YRab5
YRab7
YRab11
YRab35
e
Virgin
Mated 24h
L
Apical
Basal
L

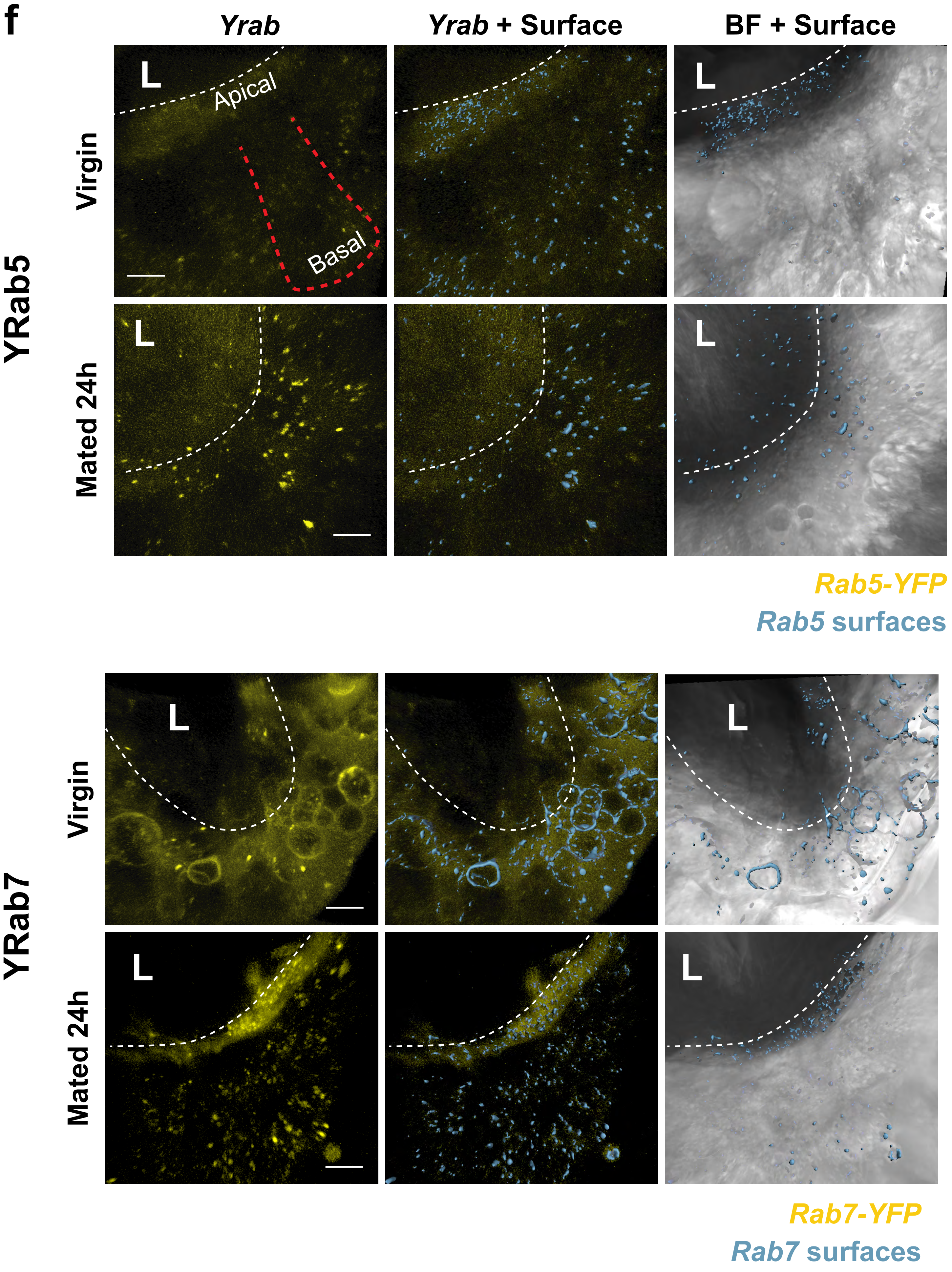

f
Yrab
Yrab + Surface
BF + Surface
YRab5
Virgin
Mated 24h
L
Apical
Basal
Rab5-YFP
Rab5 surfaces
YRab7
Rab7-YFP
Rab7 surfaces

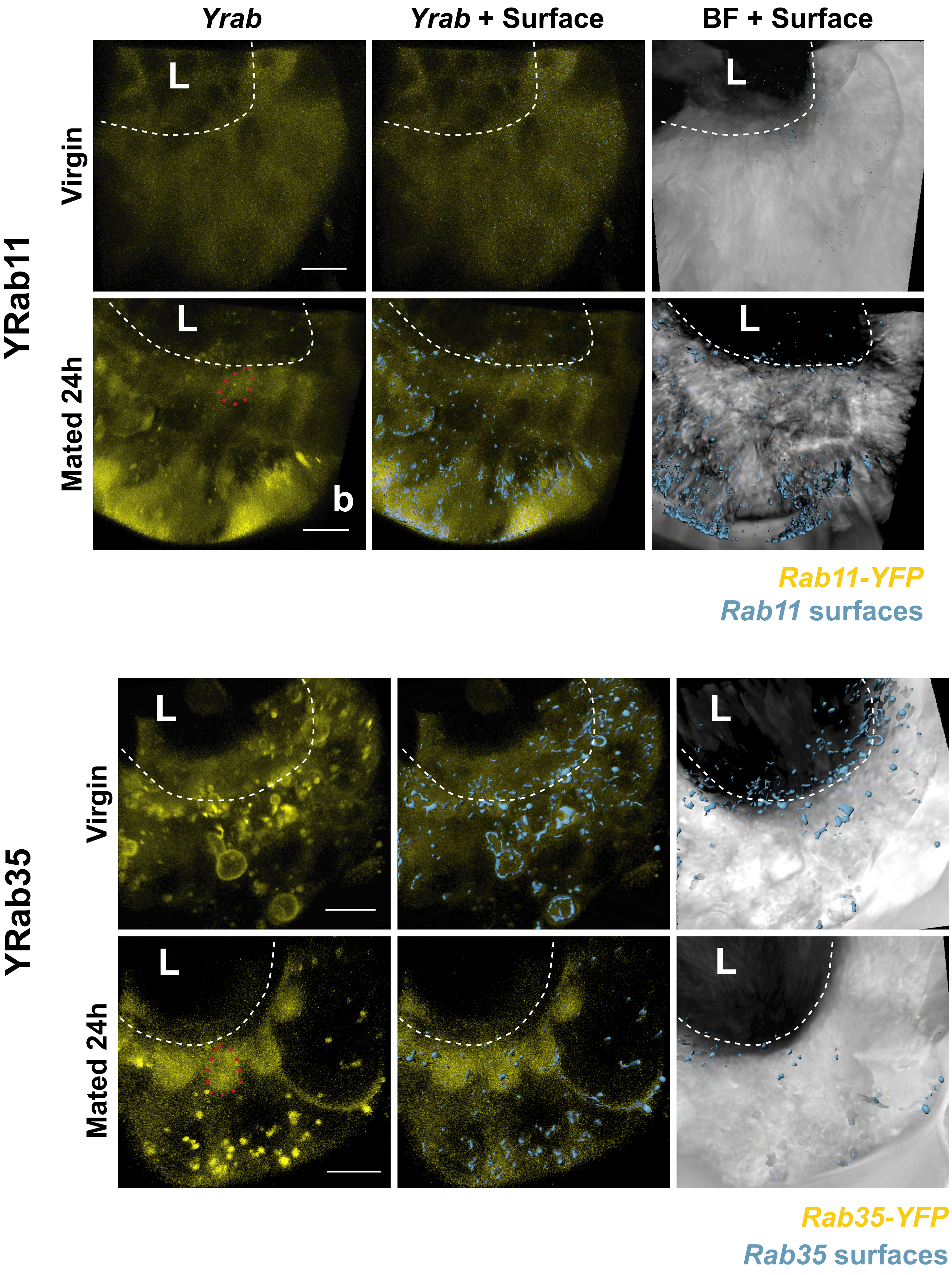

Yrab
Yrab + Surface
BF + Surface
YRab11
Virgin
Mated 24h
L
b
Rab11-YFP
Rab11 surfaces
YRab35
Rab35-YFP
Rab35 surfaces

Supplementary Fig. 2. SSC are enriched in Rab GTPases and their expression level and subcellular localization is modulated by mating.

a) Expression of small GTPases (IPR001806) in the spermatheca. The heatmap presents (left to right): **Spt** - expression level of genes in virgin female spermatheca; **Spt/WF** - expression of genes in the spermatheca relative to the Whole female; expression level of genes in the spermatheca of **Virgin**, and mated females at 3 **(M3h**) and 6 hours post-mating (**M6h**) (mean subtracted; log2, based on Prokupek et al datasets[20]). **b**) Schematic that describes the analysis of YRab localization in the spermatheca. The grid was laid over the maximum projection images of the spermathecae, adjusting over the lumen and borders, and divided to 8 radial sections. The different subcellular regions (apical, medial and basal; see also **Fig. 2a**) were quantified to estimate the YRab patterns of expression for each section (7 sections, excluding the section of the stalk). **c)** Expression profiles of different YRab patterns in the subcellular regions of virgin and mated at 24h post-mating SSC (see also **Fig. 2a**). The heatmap color code represents the percentage of regions in the spermatheca where a specific YRab was detected in low (1) or high (2) abundance (for YRabs associated with punctate, and large membranal compartment LMC pools), or present (1) or absent (0) from the region of analysis (YRabs associated with diffuse pool), out of the total regions in which YRab was observed (the number of regions expressing YRabs is shown at the bottom of each YRab heatmap). To compare proportions, we applied the two-proportion comparison test corrected by the Benjamini-Hochberg correction for multiple comparisons[77] based on ranked *p*-values, in which the maximum allowed *p* was 0.20. **d)** Cartoon representations summarizing the YRab profiles found in **c** and **Fig. 2c**. **e)** Representative confocal maximum projections of whole spermathecae expressing the different YRabs; broken red line highlight the SSC; scale bar = 20µm. **f**) Magnification of spermathecae in which a specific YRab is visualized in the SSC. The YRab signal was rendered as blue surfaces in Imaris and overlaid on the bright field to highlight their subcellular localization within the SSC. Red broken lines indicate SSC, and white lines, the apical subcellular region lining the spermatheca lumen (L). In YRab11 and YRab35 the red spotted lines delineate end apparatus. (BF) – bright field; scale bar = 5µm.

Fig. S3

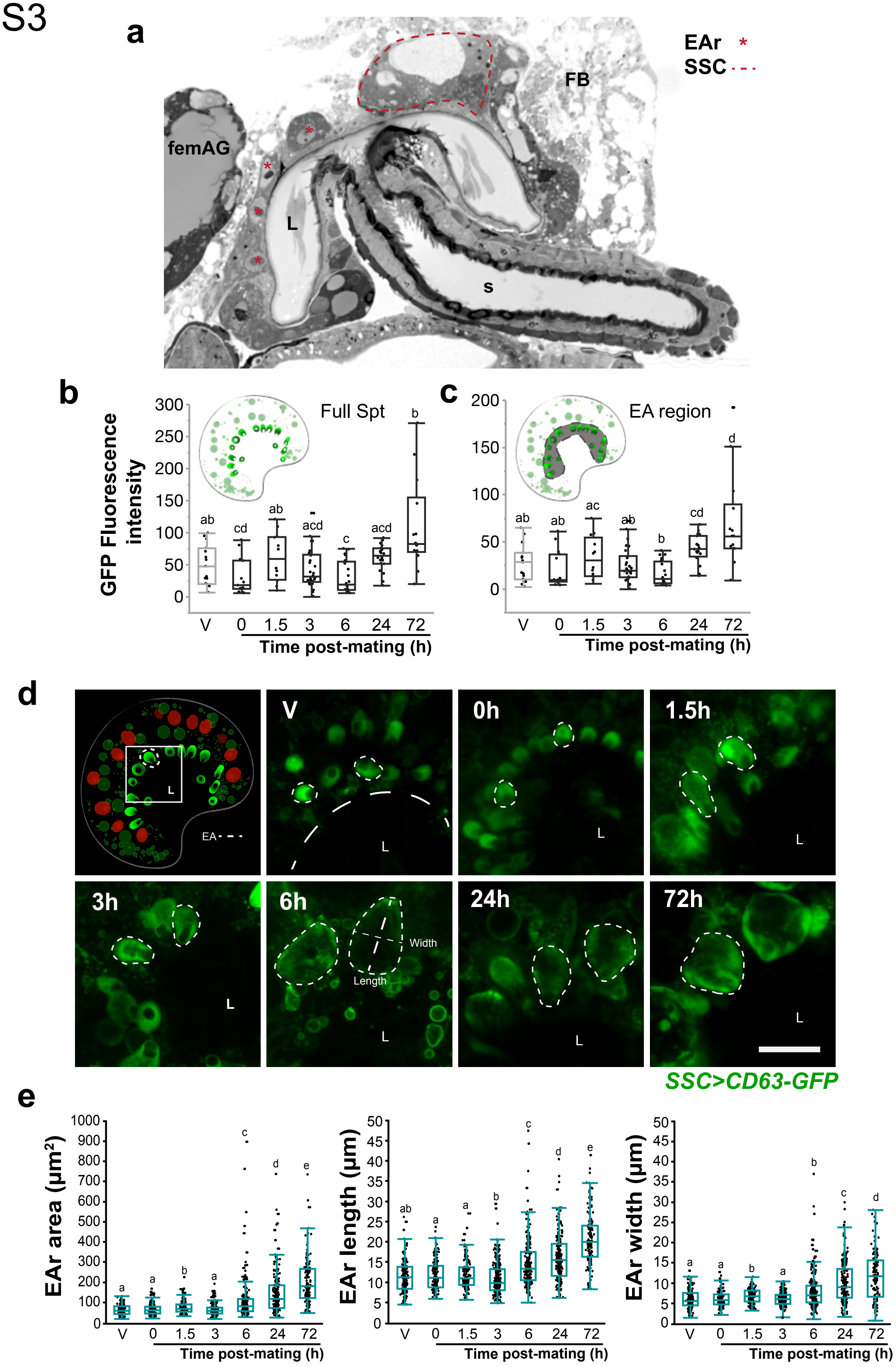
a
EAr *
SSC
FB
femAG
L
s
b
GFP Fluorescence intensity
Full Spt
V
0
1.5
3
6
24
72
Time post-mating (h)
c
EA region
Time post-mating (h)
d
EA
V
0h
1.5h
3h
6h
Width
Length
24h
72h
L
SSC>CD63-GFP
e
EAr area (μm²)
EAr length (μm)
EAr width (μm)
Time post-mating (h)

Supplementary Fig. 3. The end apparatus undergoes changes post-mating plausibly to support secretory activity of the spermatheca.

The end apparatus reservoir (EAr) collects the SSC secretions and it is formed by the invagination of the apical SSC membrane over a fibrillar cuticular meshwork that connects the secretory cavity to the lumen through a cuticular duct[17,78] (see **Fig. 1b**). **a**) Toluidine blue stained 1-µm thick section of virgin female spermatheca: spermatheca secretory cell (SSC; broken line), EAr (star), lumen (L), stalk (s), female accessory gland (femAG), and fat body (FB) [imaged by Pat Rivlin, Anat Kapelnikov, Ron Hoy and Yael Heifetz]. **b**) CD63-GFP fluorescence intensity level of the whole spermatheca, and **c**) end apparatus (EA) region of the spermatheca as shown in the small schematics above (grey outline; in continuous to **Fig. 3a** and **b**). Box plots represent maximum, median and minimum values with outliers of 15-20 spermathecae per condition; letters denote significant differences (one-way ANOVA, multiple comparison post-hoc test, $p<0.05$). **d**) Representative confocal micrographs (from the region shown in the inset of the schematic) displaying morphological changes that the EA undergo in virgin and mated females (0, 1.5, 3, 6, 24 and 72h post-mating) SSC; L= lumen; scale bar= 5µm (see **Fig. 1b**). **e**) Morphological changes of EAr were evaluated by calculating the area (μm^2), length and width (µm) in each condition. Box plots of 10 different EAr from 30-40 spermathecae per condition, between 16-22 flies; boxes represent maximum, median and minimum values with outliers; letters denote significant differences (one-way ANOVA, multiple comparison post-hoc test, $p<0.05$).

Fig. S4

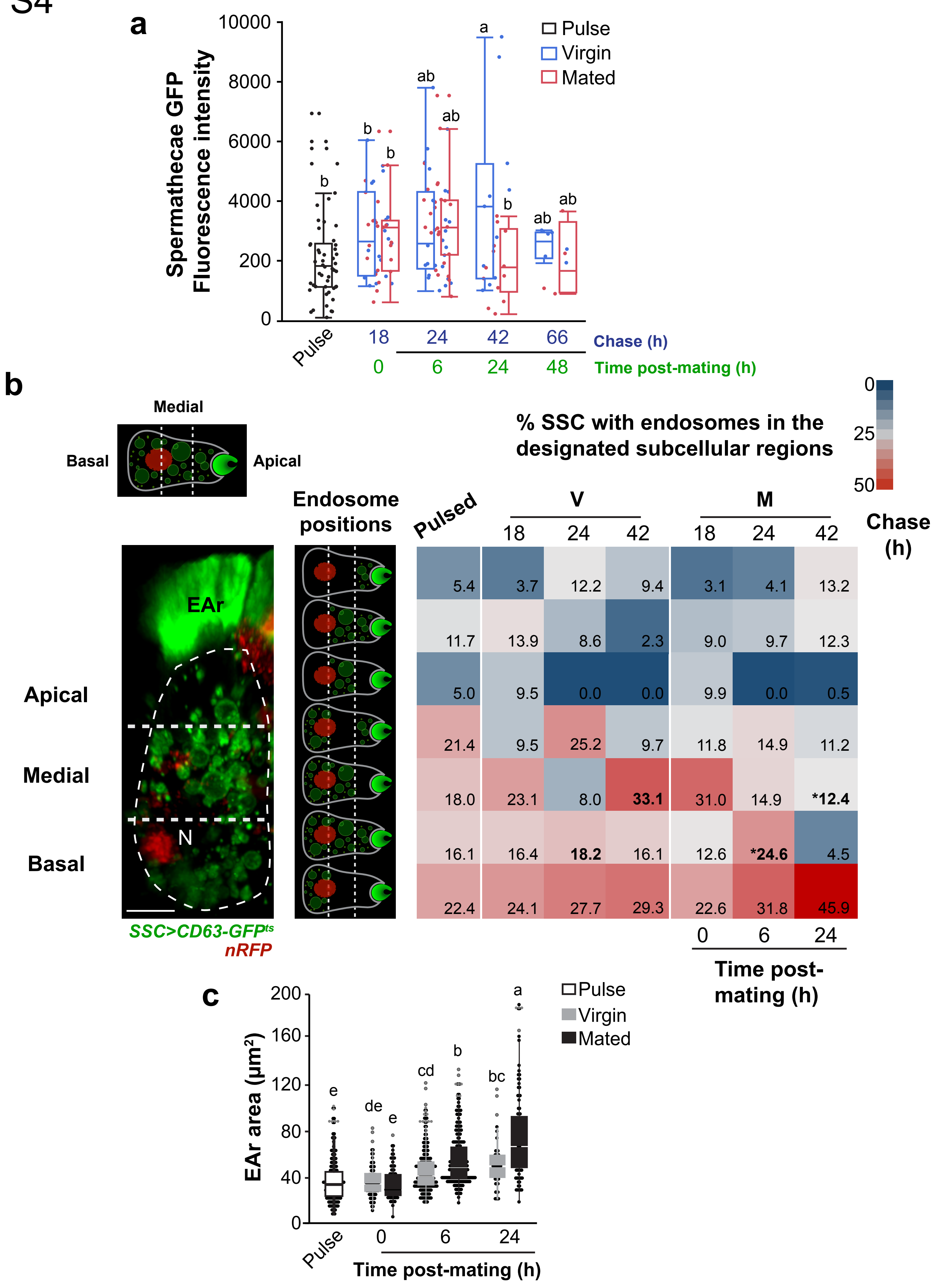
a
Spermathecae GFP Fluorescence intensity
10000
8000
6000
4000
200
0
Pulse
Virgin
Mated
a
ab
ab
b
b
b
b
ab
ab
Pulse
18
24
42
66
Chase (h)
0
6
24
48
Time post-mating (h)
b
Medial
Basal
Apical
% SSC with endosomes in the designated subcellular regions
0
25
50
Endosome positions
Pulsed
V
M
18 24 42
18 24 42
Chase (h)
EAr
Apical
Medial
Basal
N
SSC>CD63-GFP[ts]
nRFP
5.4 3.7 12.2 9.4 3.1 4.1 13.2
11.7 13.9 8.6 2.3 9.0 9.7 12.3
5.0 9.5 0.0 0.0 9.9 0.0 0.5
21.4 9.5 25.2 9.7 11.8 14.9 11.2
18.0 23.1 8.0 33.1 31.0 14.9 *12.4
16.1 16.4 18.2 16.1 12.6 *24.6 4.5
22.4 24.1 27.7 29.3 22.6 31.8 45.9
0 6 24
Time post-mating (h)
c
EAr area (µm²)
200
160
120
80
40
0
Pulse
Virgin
Mated
e
de
e
cd
b
bc
a
Pulse
0
6
24
Time post-mating (h)

Supplementary Fig. 4. The abundance of CD63-GFP-positive endosomes in the SSC increases post-mating.

CD63-GFP was conditionally expressed in the SSC by using a temperature sensitive Gal80 (CD63-GFP). **a**) GFP fluorescence intensity level was measured in the whole spermatheca of virgin and mated females to detect the dynamics of CD63-GFP degradation throughout the chase (see schematic in **Fig. 4a**). **b**) Heatmap depicting the percentage of SSC with CD63-GFP-positive end apparatus reservoirs (EAr), and endosomes in the apical, medial or basal subcellular regions of the SSC across time and mating status. The schematic columns at the left show endosome spatial distribution in the SSC. Stars and bold numbers in the heatmap depict significant differences between virgin and mated at a given time. The left image is a representative lightning confocal micrograph of an SSC with endosomes distributed in the three regions of the cytoplasm, where green is CD63-GFP and red nRFP. Scale bar = 5µm. **c**) Mean EAr area (µm^2) measured across time and mating status. Mating induced a progressive increase in the CD63-GFP-positive EAr area as observed in **Supplementary Fig 3b**. Box plots represent maximum, median and minimum values with outliers; letters denote significant differences (two-way ANOVA and multiple comparison Tukey HSD post-hoc test, $p<0.05$).

Fig. S5

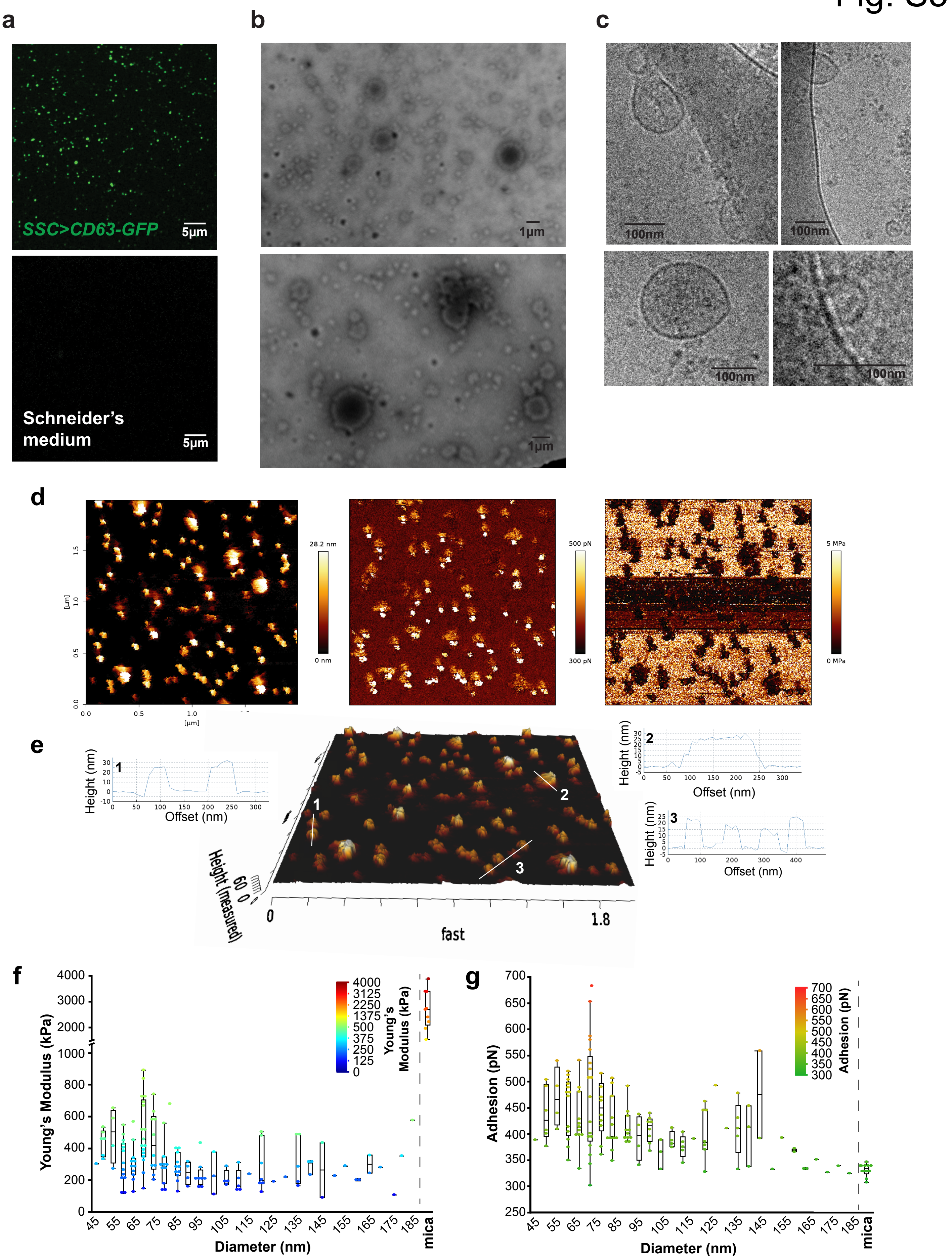

a
b
c
SSC>CD63-GFP
5µm
Schneider's medium
5µm
1µm
1µm
100nm
100nm
100nm
100nm
d
28.2 nm
0 nm
500 pN
300 pN
5 MPa
0 MPa
[µm]
e
Height (nm)
Offset (nm)
1
2
3
fast
Height (measured)
f
Young's Modulus (kPa)
Diameter (nm)
Young's Modulus (kPa)
mica
g
Adhesion (pN)
Diameter (nm)
Adhesion (pN)
mica

Supplementary Fig. 5. Characteristics of CD63-GFP SSC and spermatheca-derived-EVs. a-c) Spermathecae of females expressing CD63-GFP in the SSC were cultured *ex vivo* 72h post-mating and the spent media was assessed by different microscopy techniques (from 100 spermathecae and 52-55 flies): **a**) Spent or clean media were imaged by confocal microscopy using the Leica Lightning mode; scale bar = 5µm; **b**) The morphology of EVs in the spent media was observed by negative staining and STEM; scale bar = 1µm and 200 nm; and by **c)** CryoTEM; Scale bar = 100 nm. Shown are EVs of various sizes and morphology of the lipid bilayers. **d-g**) The spent media of *ex vivo* cultured spermathecae were imaged by AFM for the presence of EVs. **d)** Representative AFM scans (2x2µm) of EVs from spermathecae spent media (from 200 spermathecae from 105-110 flies) isolated by acoustic sorting (AcouSort). From left to right: height (µm), adhesion (pN) and Young's modulus (kPa) images. **e**) 3D profile of (**d**), exemplifying 3 cross sections of EVs (height and diameter in nm). Profiles of (**f**) Young's modulus and (**g**) adhesion force of EVs of different diameters and the mica substrate (see also **Fig. 5f** for examples of single EV AFM scans).

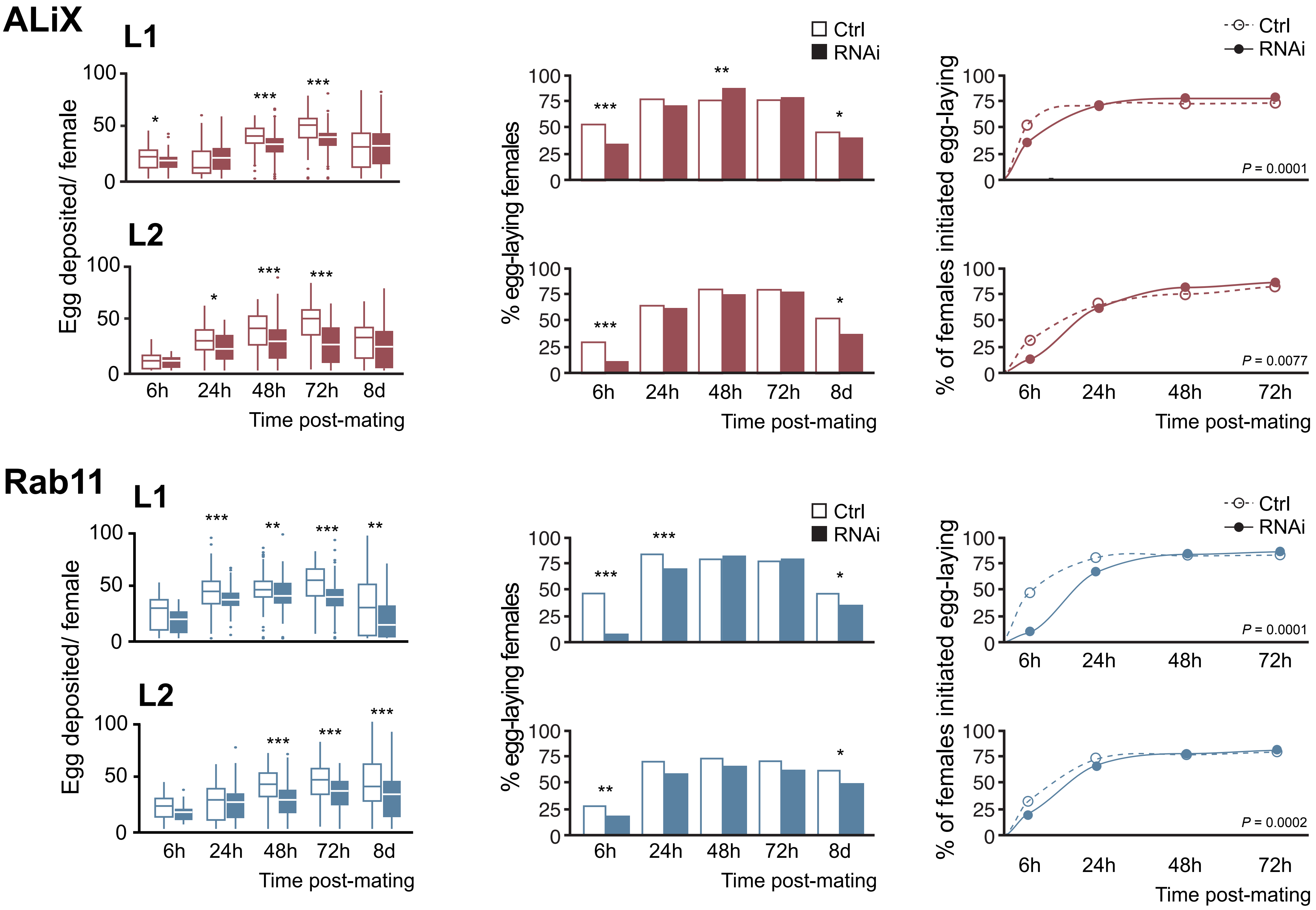
ALiX
L1
L2
Rab11
Egg deposited/ female
% egg-laying females
% of females initiated egg-laying
Ctrl
RNAi
6h
24h
48h
72h
8d
Time post-mating
P = 0.0001
P = 0.0077
P = 0.0001
P = 0.0002

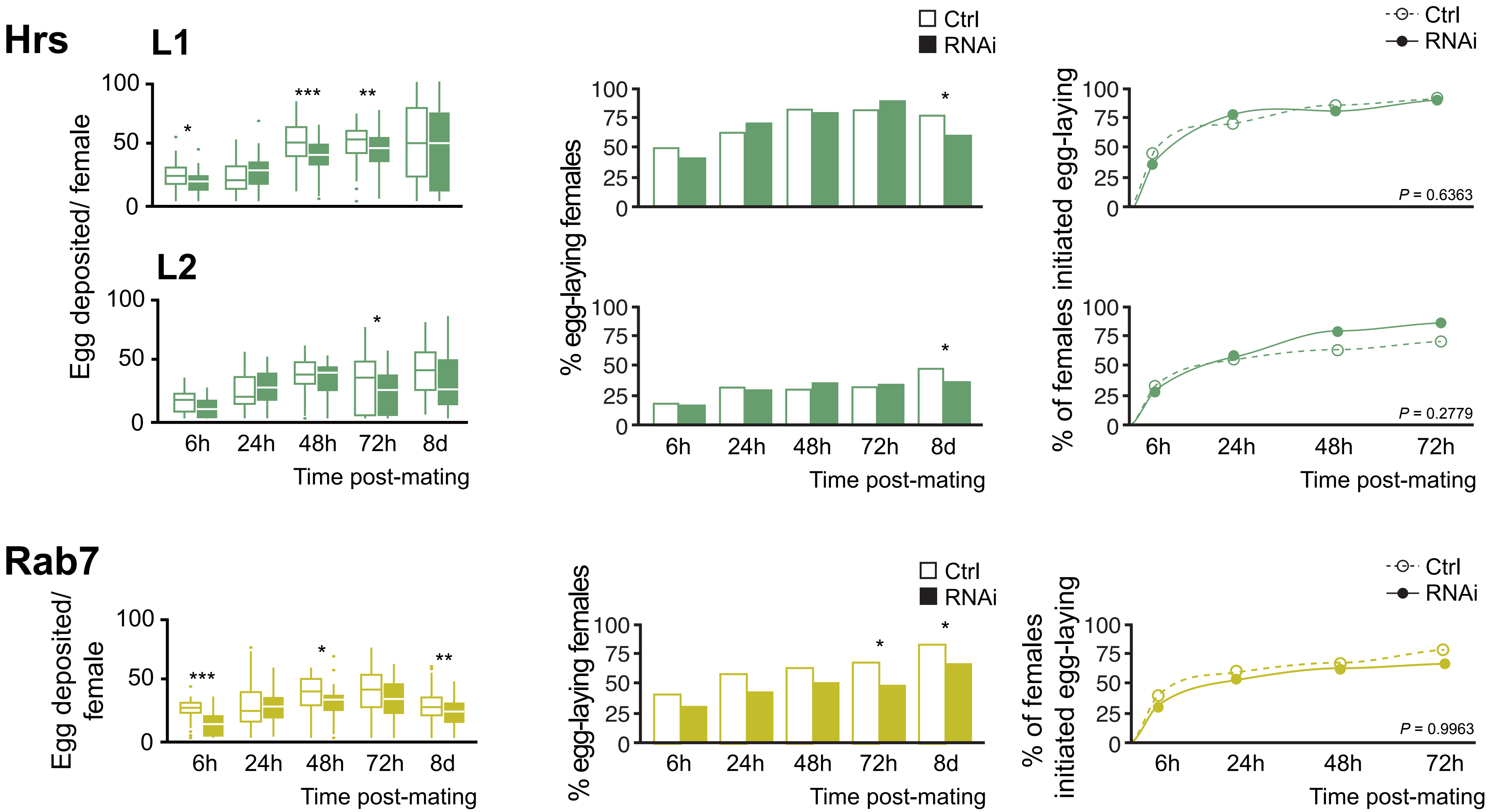

Hrs
L1
L2
Egg deposited/ female
Time post-mating
% egg-laying females
% of females initiated egg-laying
Ctrl
RNAi
P = 0.6363
P = 0.2779
6h
24h
48h
72h
8d
Rab7
Egg deposited/ female
% egg-laying females
% of females initiated egg-laying
P = 0.9963

Supplementary Fig. 6. Silencing of *ALiX*, *Rab11*, *Hrs* and *Rab7* in the SSC decreases egg-laying and delays the onset of deposition.

Following silencing of *ALiX*, *Rab11*, *Hrs* and *Rab7*, specifically in the SSC we evaluated: the number of eggs deposited per female, the percentage of females that laid eggs at each time point, and the percentage of females that initiated egg-laying (time at which the female laid her first eggs) at 6, 24, 48, 72 h and 8 days post-mating. Control females (no RNAi, open bars); RNAi expressing females (full bars). The effects of silencing were evaluated in two independent different RNAi fly lines (L1, L2) for all the genes but Rab7. Box plots represent maximum, median and minimum values with outliers, and bars and points in the curves are the percentages of flies in each category. Sample sizes: *ALiX*-RNAi (n = 87-139), *Hrs*-RNAi (n = 52-97), *Rab11*-RNAi (n = 122-153), *Rab7*-RNAi (n = 71-77). *, $P<0.05$; **, $P<0.01$; ***, $P<0.001$ (see supplementary methods for statistics).

Fig. S7

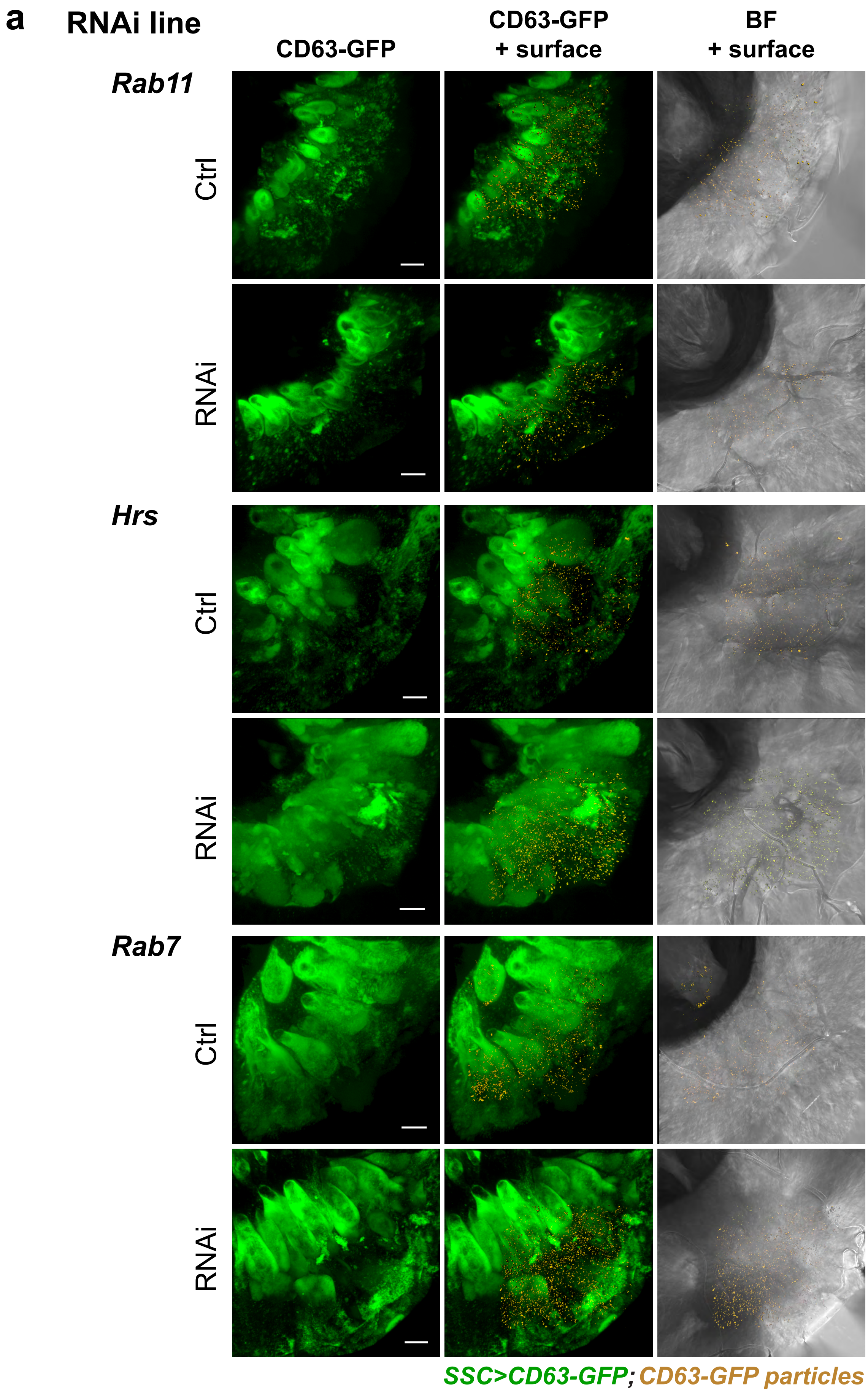
a
RNAi line
CD63-GFP
CD63-GFP
+ surface
BF
+ surface
Rab11
Ctrl
RNAi
Hrs
Ctrl
RNAi
Rab7
Ctrl
RNAi
SSC>CD63-GFP; CD63-GFP particles

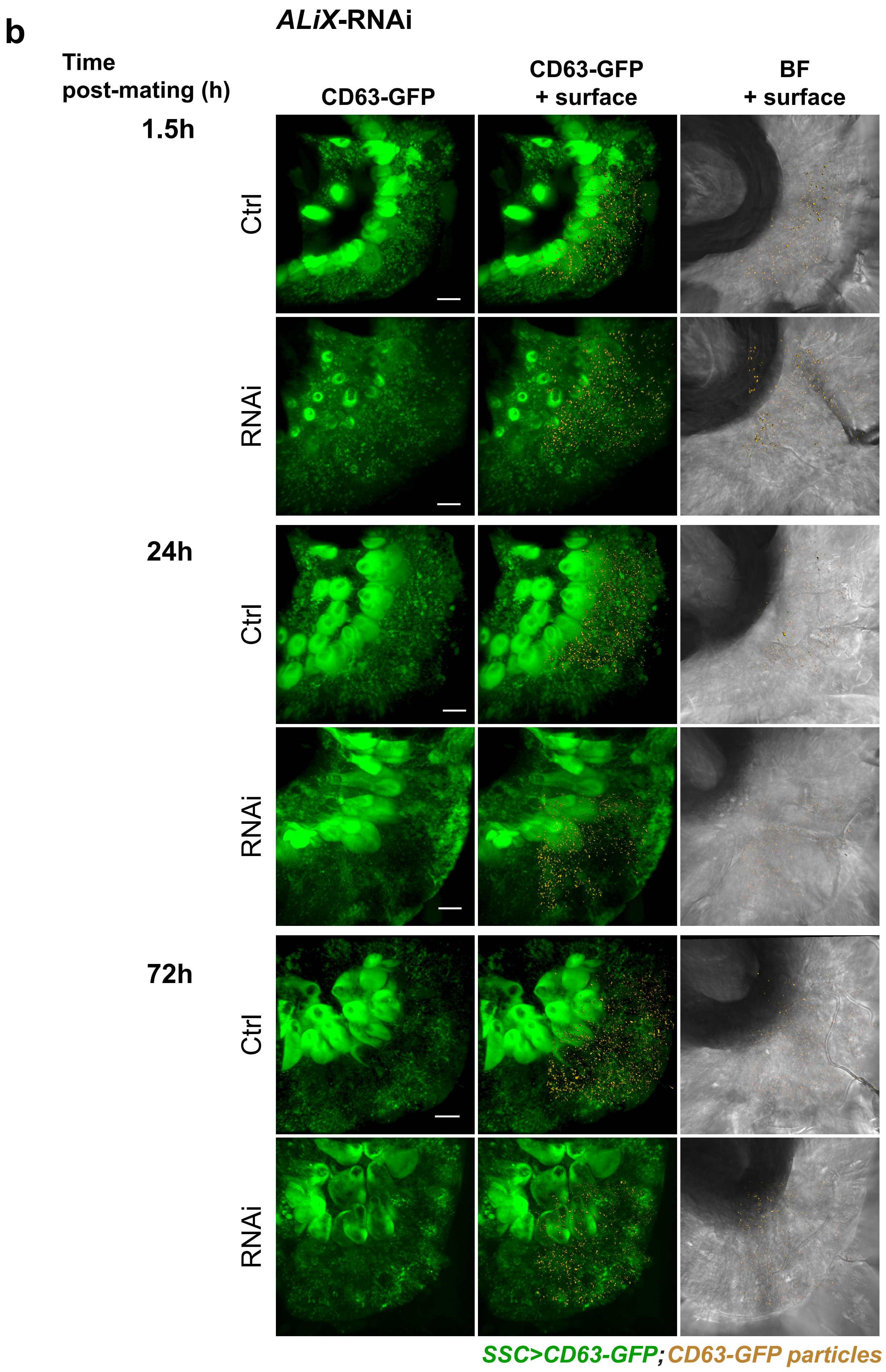
b
ALiX-RNAi
Time
post-mating (h)
CD63-GFP
CD63-GFP
+ surface
BF
+ surface
1.5h
Ctrl
RNAi
24h
Ctrl
RNAi
72h
Ctrl
RNAi
SSC>CD63-GFP; CD63-GFP particles

Supplementary Fig. 7. The intracellular CD63-GFP in the SSC is affected by mating and silencing of *ALiX*, *Hrs*, *Rab11* and *Rab7*.

Representative images of the analyzed regions of spermathecae that expressed CD63-GFP (control, Ctrl) or co-expressing RNAi and CD63-GFP for *ALiX*, *Hrs*, *Rab11* and *Rab7* (RNAi) (The sampled region is shown in the inset of **Fig. 3a**). **a**) Representative images of control, *Rab11*-, *Hrs*- and *Rab7*-RNAi, SSC at 24h post-mating. The left column demonstrates the pattern of GFP-positive puncta localized in the cytoplasm of the SSC, the middle column shows CD63-GFP particles rendered as yellow plastic surfaces using Imaris, and the right column depicts the distribution of CD63-GFP yellow surfaces in the spermatheca at brightfield (BF) for spatial reference. **b**) Representative images of the CD63-GFP expression in the spermathecae of control and *ALiX*-RNAi, at 1.5, 24 and 72h post-mating. See also **Fig. 6c and d** where the number and mean particle size are evaluated. Scale bar= 10µm.
