# Supplementary material for: Male-female communication enhances release of extracellular vesicles leading to high fertility in Drosophila
Source: Commun Biol. 2022 Aug 13;5:815. doi: 10.1038/s42003-022-03770-6 (PMC9376107; doi:10.1038/s42003-022-03770-6)
Supplement: Supplementary file 2 — Description of Additional Supplementary Files [file 42003_2022_3770_MOESM2_ESM.pdf]

**Description of Additional Supplementary Files**

**Supplementary Movies**

**File name:** Supplementary Movie 1. Live SSC activity.
**Description:** This movie demonstrates how the endosomal and end apparatus activity vary between virgin and 72h post-mating spermatheca imaging live SSC that express CD63-GFP (as shown in **Fig. 3a**). The imaged section of the spermathecae depicts a single layer of SSC. The arrow in the 72h movie points at a multivesicular body-like endosome that displays intraluminal CD63-GFP puncta. Each stack was captured every minute for 30 min. Scale bar = 10µm.

**File name:** Supplementary Movie 2. SSC displaying endosomal activity.
**Description:** The movie demonstrates live SSC expressing CD63-GFP, stained with LysoTracker Deep Red. In the cytoplasm next to the end apparatus reservoir, the arrow points at a large endosome that displays active motion of CD63-GFP punctated structures which might represent intraluminal vesicles. Each stack was captured every 7 seconds for 10 minutes. Scale bar = 5µm.

**File name:** Supplementary Movie 3. Overview of the spermatheca lumen with CD63-GFP EVs.
**Description:** This movie navigates through the 3D projection of a spermatheca expressing CD63-GFP in the SSC (**Fig. 3e**). The overlay of the fluorescence on the bright-field allows the visualization of CD63-GFP EVs found in the lumen of the spermatheca where the sperm is stored. Scale bar = 20µm.

**File name:** Supplementary Movie S4. Overview of the spermatheca lumen with myr-RFP EVs.
**Description:** This movie navigates through the 3D projection of a spermatheca expressing myr-RFP in the SSC (**Fig. 3f**). The overlay of the fluorescence on the bright-field allows the visualization of myr-RFP EVs in the lumen of the spermatheca where the sperm is stored. The scale bar size changes as the video progresses.

**File name:** Supplementary Movie 5. Activity of spermatheca in *ex vivo* culture.
**Description:** This movie depicts the activity of spermatheca expressing CD63-GFP in the SSC at 72h post-mating. The spermathecae are cultured in Schneider's media alone or with 10µM Ecdysone capturing a stack every 3 min over a lapse of 75 min. Scale bar = 20µm.

**File name:** Supplementary Movie 6. Sperm stored in the spermatheca lumen surrounded with myr-RFP EVs.
**Description:** This movie navigates through the 3D projection of a spermatheca expressing myr-RFP in the SSC (**Fig. 8c**). The overlay of the fluorescence on the bright-field allows the visualization of sperm heads (DAPI) in proximity to myr-RFP EVs in the lumen of the spermatheca. The scale bar size changes as the video progresses.

**Supplementary Data**

**File name:** Supplementary Data 1.
**Description:** Dataset of the expression of genes in the spermatheca.

**File name:** Supplementary Data 2.
**Description:** Source data of all experiments.
